# Uganda’s response to sexual harassment in the public health sector: from “Dying Silently” to gender-transformational HRH policy

**DOI:** 10.1186/s12960-021-00569-0

**Published:** 2021-05-01

**Authors:** Constance Newman, Alice Nayebare, Stella Neema, Allan Agaba, Lilian Perry Akello

**Affiliations:** 1 IntraHealth International, 6340 Quadrangle Drive, Suite 200, Chapel Hill, NC 27510 USA; 2 Formerly an employee of Intrahealth International, Kampala, Uganda; 3 Department of Sociology and Anthropology. School of Social Sciences, Makerere University, P. O. Box 7062, Kampala, Uganda; 4 Uganda Ministry of Health, Plot 6, Lourdel Road, Wandegeya, P.O Box 7272, Kampala, Uganda

**Keywords:** Sexual harassment, Gender-transformative workforce policy, Support supervision, Performance and human resources management

## Abstract

**Introduction:**

Sexual harassment is a ubiquitous problem that prevents women’s integration and retention in the workforce. Its prevalence had been documented in previous health sector studies in Uganda, indicating that it affected staffing shortages and absenteeism but was largely unreported. To respond, the Ministry of Health needed in-depth information on its employees’ experiences of sexual harassment and non-reporting.

**Methods:**

Original descriptive research was conducted in 2017 to identify the nature, contributors, dynamics and consequences of sexual harassment in public health sector workplaces and assess these in relation to available theories. Multiple qualitative techniques were employed to describe experiences of workplace sexual harassment in health employees’ own voices. Initial data collection involved document reviews to understand the policy environment, same-sex focus group discussions, key informant interviews and baseline documentation. A second phase included mixed-sex focus group discussions, in-depth interviews and follow up key informant interviews to deepen and confirm understandings.

**Results:**

A pattern emerged of men in higher-status positions abusing power to coerce sex from female employees throughout the employment cycle. Rewards and sanctions were levied through informal management/ supervision practices requiring compliance with sexual demands or work-related reprisals for refusal. Abuse of organizational power reinforced vertical segregation, impeded women’s productive work and abridged their professional opportunities. Unwanted sexual attention including non-consensual touching, bullying and objectification added to distress. Gender harassment which included verbal abuse, insults and intimidation, with real or threatened retaliation, victim-blaming and gaslighting in the absence of organizational regulatory mechanisms all suppressed reporting. Sexual harassment and abuse of patients by employees emerged inadvertently.

**Discussion/conclusions:**

Sex-based harassment was pervasive in Ugandan public health workplaces, corrupted management practices, silenced reporting and undermined the achievement of human resources goals, possibilities overlooked in technical discussions of support supervision and performance management. Harassment of both health system patients and employees appeared normative and similar to “sextortion.” The mutually reinforcing intersections of sex-based harassment and vertical occupational segregation are related obstacles experienced by women seeking leadership positions. Health systems leaders should seek organizational and sectoral solutions to end sex-based harassment and make gender equality a human resource for health policy priority.

**Supplementary Information:**

The online version contains supplementary material available at 10.1186/s12960-021-00569-0.

## Introduction

### Background

Sexual harassment is a ubiquitous problem that prevents women’s integration and retention in the workforce [1–3]. The prevalence of sexual harassment in Uganda’s public health sector had been documented and linked to staffing shortages and absenteeism. In a 2003 Uganda Ministry of Health (UMOH) study on health worker retention, around 24% of workers the majority of whom were female nurses reported that they had been subjected to sexual “abuse” by a supervisor [4]. Approximately one in five reported sexual abuse by patients or their relatives (21%). Fewer reported abuse by peers (16%) or while travelling to and from work (18%). Health workers reported quitting in response to such abuse.

A 2012 UMOH descriptive gender research study, Gender Discrimination and Inequality Analysis [GDIA [5] found that men were overwhelmingly concentrated in senior levels of management. About 32% of GDIA survey respondents reported that manager/supervisor expectations of sexual favors in exchange for a good evaluation, a promotion, or a salary raise (i.e., *quid pro quo* sexual harassment) were either “somewhat common” or “very common.” Focus group (FGD) respondents perceived gendered power and subordination (“*When men are bosses, they think they can take anything they want from female subordinates, so they start asking for sexual favors”*) and retaliation (e.g., a woman who “stands her ground” runs the risk of a bad evaluation or job loss). FGD participants affirmed that *“Some decide to ignore it while others suffer quietly”* while others quit their jobs or found different ways of coping *rather than report* it (“*Sexual harassment is silent; no one discloses*).” District managers confirmed that rampant sexual harassment was a *“serious form of corruption.”* Other forms of sexual harassment included 1) sexually suggestive gestures (30%); 2) being exposed to sexually explicit discussions or conversations (25%); 3) unwanted attempts to establish sexual relationships (22%); and 4) being the object of sexual jokes, comments, or leering (19%). There were also co-occurring stereotypes of women’s leadership incompetence and discrimination based on pregnancy and family responsibilities, which sidelined female health workers.

In summary, sexual harassment, especially by supervisors, was a silent and apparently unregulated problem in Uganda’s public health workplaces, mainly affecting female employees, co-occurring with other types of gender discrimination, in a context of vertical segregation. Other Ugandan studies, while measuring the prevalence of sexual harassment in different ways, found it to be a problem in other public sectors, from Parliament [6], the police force [7], the prison system [8], education [9], to agriculture and at public markets, where men’s non-consensual touching of women is so common as to have a name– *bayeye* [10]. While Uganda had launched national *Sexual Harassment Regulations* in 2012, their implementation was uneven.

## Methods

Prevalence had been previously measured, but information on how to prevent and respond to sexual harassment was lacking. Not everything that counts can be counted [11], so additional UMOH research in 2017 involved a new descriptive approach employing multiple qualitative data collection techniques to address key questions: *What are UMOH employees’ experiences of sexual harassment? Why is non-reporting of sexual harassment pervasive? What are the consequences? What is the cross-cultural relevance of current theories, definitions and dynamics for UMOH human resources for health policy and human resources management (HRM)?* The 2017 research not only aimed to describe the workings of sexual harassment and non-reporting in UMOH workplaces but also assessed the relevance of current sexual harassment theory and definitions, at a minimum, to increase the consistency of measurement across settings. This approach required balancing discovery of participants’ lived experience with the explanatory potential of pre-existing categories of understanding from the literature. The literature informed data collection tools and interpretation.

### Relevant literature

UMOH stakeholders stressed the importance of anchoring the research in the framework of Uganda’s *Sexual Harassment Regulations.* The literature review (published and “gray”) examined theories that account for sexual harassment, how it is defined, and the problems and dynamics of reporting.

### Theories

There is no single explanation or theoretical framework that fully accounts for sexual harassment. Some include [12, 13]:

- Nature: Sexual harassment is a natural extension of mate selection in evolutionary theory, behavior that is motivated by mutual sexual desire and “natural” and therefore not a social or workplace problem.
- Sex role/sociocultural spillover: Sexual harassment is the outcome of norms that socialize men into sexual assertion, social dominance and superiority, and persistence, and women into sexual submission and passivity. Harassers bring inappropriate expectations to workplace interactions, thereby perceiving women in their sex, rather than their work, roles. The result of these inappropriate role expectations is behavior that is perceived as sexually harassing.
- Economics: Sexual harassment is used to drive out women (and cultural minorities) who compete for valued jobs traditionally held by men, as if to communicate “women don’t belong here” [14]. Maintaining the most highly-rewarded forms of work as domains of masculine competence results from “tormenting members of minority and other disadvantaged groups seeking upward mobility through work” [15].
- Power: Organizations are “public patriarchies” in which *violence and violences* are forms of power, domination and oppression that structure organizations [16, p.20 and p.29]. Sexual harassment embodies sexist ideologies of male superiority and female inferiority. The (patriarchal) gender order of the larger society translates into organizational gender regimes [17] which are maintained through a dominant form of [hegemonic] masculine behavior *vis a vis* ideal feminine behavior to maintain an unequal gender order. Power differentials increase the likelihood of sexual harassment, and since men typically hold more power, they are more likely to be perpetrators [18]. Women are at most risk to be targets of sexual harassment and more vulnerable to its economic, psychological, social and physical consequences. Managers and supervisors (in the health workforce, predominantly men [19]) are structurally situated in organizational hierarchies to exercise power [18] over their subordinates and have control over work-related outcomes such as positive performance evaluations, salary increases, or flexible work [18].

There is a substantial vein of research and human rights literature that puts unequal power at the center of theory where asymmetries of power [20], threats or acts of violence, gender stereotyping and economic control of work result in the subordination of women. Cockburn’s early research led her to conclude that women’s presence in the workplace was a highly political issue for men, and that women’s claim to economic independence and an equal place in organizational life called forth new measures of exclusion and reassertion of male authority [21, p. 143]. Sexual harassment maintains an already existing gender stratification through “the unwanted imposition of sexual requirements in a relationship of unequal power” [22]. While men may be vulnerable to harassment if they are perceived as feminine, women are targeted when they challenge their subordinate position in the gender order. Konik and Cortina augmented *sex-based* harassment theory by studying sexualized harassment, gender harassment, and heterosexist harassment, which yield an integrated model of workplace oppression based on gender-role enforcement, i.e., “policing gender” [23]. In Schultz’s thinking, the problem with workplace harassment is sexism and not sex; it is more about upholding gendered status and identity than it is about expressing sexual desire [24].

The United Nations posited “that violence against women (including sexual harassment) is a manifestation of historically unequal power relations between men and women, which have led to domination over and discrimination against women by men and to the prevention of the full advancement of women, and that violence against women is one of the crucial social mechanisms by which women are forced into a subordinate position compared with men” [25].

The term "violence against women" means any act of gender-based violence that results in, or is likely to result in, physical, sexual or psychological harm or suffering to women, including threats of such acts, coercion or arbitrary deprivation of liberty, whether occurring in public or in private life [25]. A COFEM brief recently noted that “everyday sexism, sexual harassment and other forms of gender-based violence share a root cause—gender inequality and the oppression of women and girls– and distinguishing different forms of gender-based violence as more serious than others ignores how patriarchy and gender inequality create a culture in which [all] violence against women and girls is accepted and normalized” [26].

Summarizing power theories, sexual harassment is an assertion of power, a manifestation of historically unequal power relations between men and women, serves to police appropriate ways of “doing gender,” penalizes gender non-conformity [27] and protects or enhances men’s gender-based social status [24]. The 2017 UMOH research assessed the cross-cultural relevance of these theories. It should be noted that patterns of workplace sexual harassment of men may differ from the sexual harassment of women and sexual minorities. The reader is therefore directed to relevant readings [28–31].

### Definitions

Fitzgerald, Berdahl, Schultz and Leskinen et al. [24, 27, 32–36] are researcher-theorists whose definitions reflect the gender inequality and power concerns of “sex-*based* harassment” which encompasses *sexual coercion, unwanted sexual attention* and *gender harassment*, the latter involving hostile behaviors that are believed to have little to do with sexuality, and everything to do with gender [33]. Sexual coercion and unwanted sexual attention include behaviors consistent with the well -known categories *“quid pro quo”* and *hostile environment* harassment; the third category, *gender harassment*, includes a wide range of sexist, insulting, demeaning behaviors that are motivated by hostility toward individuals who violate gender ideals or norms, not by desire for those who meet them [27]. Gender harassment does not typically aim at sexual cooperation and is “more put down than come on” [33]. This category is useful for understanding other aspects of women’s experiences in hostile work environments. For example, Leskinen and Cortina augmented the category to include sex-related insults featuring hostility against motherhood status [36]. Indeed, a broader range of harassing, discriminatory behaviors that co-occur with “sexual harassment” (e.g., pregnancy discrimination and “hostile animus”) has been documented in recent health workforce research [37–41].

Additional file 1, *Illustrative Definitions,* shows the variety of definitions of sexual harassment in the literature. It is defined as violence, discrimination, an assault on dignity or a human rights violation. Some focus on the sexual nature of “quid pro quo” and hostile environments, while others define three categories of “sex-based harassment.” Reflecting this variety, the 2019 ILO Convention 190 [42, 43] was inclusive and defined “violence and harassment” broadly, though it includes “gender-based violence and harassment”. A narrow focus on sex or conduct of a sexual nature ignores the broader range of *gender harassing and policing behaviors* that figure in gender-power theories and that occur in health workplaces [44]. A broadly inclusive definition involves challenges for measurement across settings. The UMOH research was framed by Uganda’s definition of “sexual harassment” which describes conduct of a sexual nature in *quid pro quo* and hostile environment harassment [see Additional file 2, *Definition and Interpretation from Uganda’s Employment (Sexual Harassment) Regulations, 2012*].

### Problems of reporting

There is substantial evidence of under- or non-reporting of workplace violence (which includes sexual harassment). The ILO/ICN/WHO/PSI *Framework Guidelines for Addressing Workplace Violence in the Health Sector* identified sexual harassment as a form of both physical and psychological violence [45]. Di Martino noted that, though reporting is essential for an effective response to health sector violence, reporting procedures were often lacking in his six study countries [46]. Employers did not investigate violence adequately and effective investigation did not follow, resulting in impunity. Unions, associations and the community were slow to support targets of workplace violence, and in fact, played an insignificant role in protecting their members. When targets did report incidents of violence to managers or colleagues, they were less forthcoming about sexual and racial harassment. A 2006 study of sexual harassment in the health sector of India found that 57% of doctors and nurses in the sample had been harassed, but only 29% made a formal complaint [47]. In a 2008 study of health workplace violence in Rwanda, 40% of targets disclosed to no one [38].

That sexual harassment is under/un-reported is well-established [48, 49]. A 2015 European Union survey found that out of all women who described the most serious incident of sexual harassment that had happened to them, 35% did not speak about it to anyone, only 4% talked to an employer or boss, and less than 1% consulted a lawyer, a victim support organization or a trade union representative [50]. Reasons given for non-reporting of sexual harassment appear consistent across studies: Procedural and evidentiary **(**burden of proof) hurdles; a belief that nothing will come of a report; feelings of shame and fear of being ostracized by co-workers and retaliated against for reporting; considered as a normal part of work [51]; repercussions such as being fired or blacklisted or having to quit, damage to reputation and loss of career prospects, and conflicting emotions about the harasser [52]. Retaliation is a real risk, as victims who file harassment complaints are much more likely to lose their jobs than those who experience similar levels of harassment and say nothing [53]. The targets of sexual harassment experience a range of personal, professional and organizational harms which are relevant to human resources for health and management (HRH/M), such as decreased job satisfaction and morale; increased absenteeism and job loss/leaving; deteriorating relationships with coworkers; financial stress and incremental economic harms to the employee [54], higher rates anxiety, depression and PTSD [55–57].

### Dynamics of silencing and non-reporting

It is possible that non-reporting is pervasive because of the evidentiary hurdles, for example, in documenting its psychological harms. There are also psychological and social dynamics that may explain the tendency for targets to remain silent and not report. The notion of the *rape myth* has been extensively used in sexual violence research to understand the sociocultural context of non-disclosure [58]. False cultural beliefs about the culpability of the victim and the innocence of the sexual offender, or the illegitimacy of rape as a serious offense [59] serve to deny and justify sexual aggression against women [60]. For example, “What was she wearing?” deflects responsibility onto the victim’s dress. *Rape myths* are driven by (1) gender inequality and society’s acceptance of patriarchy and male dominance, leading to tolerance of aggression against women; and (2) structural violence within which societal tolerance normalizes, justifies and legitimizes sexual violence against women, so that we do not *see* the violent act, or at least not as violence [58]. Baugh [61] found that the reason so few instances of sexual harassment are formally reported, and why so many targets who do make formal reports see the situation as worsening, is the pervasive tendencies to “blame the victim for her own plight” and to discount the target’s definition of sexual harassment. “Blaming the victim” facilitates the persistence of sexual harassment because institutionalized contributors or responses remain unquestioned [61]. Power differentials in male-dominated workplaces legitimize and institutionalize male perspectives and definitions [61].

Attempts at holding perpetrators to account for harassing, violent behavior typically evoke defensiveness and hostility [62]. In what has been described as a “DARVO” dynamic (*Deny, Attack, Reverse Victim and Offender*) [63], a perpetrator’s response to being held accountable puts the target of violence under scrutiny, and casts the target (or whistleblower) as the perpetrator. Examples include accusing the target of seeking revenge for a poor performance appraisal, or using terms like “male-bashing” [62] which suggests that it is the victim who is the violent party for bringing a charge of harassment, or for trying to affix perpetrator responsibility. *Institutional betrayal* describes organizational actions/inaction that are experienced as violations of trust that exacerbate the original harm of sexual harassment [60]. Examples include when the administration shows excessive concern for the future of the perpetrator, or when it participates in a target’s demotion, transfer or firing—effectively silencing attempts to stop the harassment. “*Gaslighting”* refers to a form of psychological manipulation in which a person or a group sows seeds of doubt and undermines self-confidence, making the target question their own memory, perception, or judgment, again with an effect of silencing [64]. Stark developed the notion of *manipulative gaslighting*, which denies, minimizes or challenges testimony about harms done to the target [65], by *sidestepping* evidence that supports the target’s testimony, or attributing cognitive or characterological defects to the target, e.g., “Can’t you take a joke? “ or “Why are you obsessing on this?” Ahern referred to *whistleblowing gaslighting*, which involves trauma resulting from the emotional manipulation used by employers to discredit and punish employees who report misconduct [66].

A reluctance to report or even label behaviors as sexual harassment is documented in academic medicine, where for example, female physicians-in-training developed strategies such as “not sweating the small stuff” (i.e., minimizing) and humor as tactics of resistance to deal with hostile environments, and acceptance of mistreatment which was normalized and passed from one generation to the next [67, p.5]. Wear and Altman also remark that physicians-training learn institutional norms that whistleblowing against one’s peers is considered unprofessional or unreliable behavior, and that gender socialization may encourage women to emphasize empathy for harassers over confrontation and punishment. Hinze [68] found that a target’s self-doubt and asking themselves if they are being “too sensitive” were common reactions in which attention was deflected back onto the target of harassment. Hinze also suggested that denying or ignoring harassment, or “not taking it personally,” are tactics used by female physicians to distance themselves from the stigma of being publically devalued, and that these tactics interrupt the naming of sexual harassment, and ensure its continuance [68]. All the foregoing processes serve to silence a target, inhibit reporting and maintain impunity.

Hearn and Parkin suggested that the recognition of violence is difficult not only because the relative isolation of survivors and feelings of shame or self-blame, but because violence and violation contradict the dominant ideological constructions of most organizations [16]. As organizations become more aware, violence is more likely to be identified, recognized, problematized, “spoken” and contested, but then is followed by further organizational “dynamics of violation.” Dynamics of violation are at play in the institutional betrayal of whistleblowers, such as official or unofficial reprisals, reprimands, punitive transfer, referral to a psychiatrist, social ostracism—all of which, the authors claim, should be anticipated. These damaging processes make it hard to keep sexual harassment “spoken” in the face of organizational pressure to silence targets [16].

### Data collection tools and strategy

The qualitative data collection techniques employed by the study elicited UMOH employees’ lived experience with or observations of sexual harassment and factors that constrained reporting. In Phase 1, data collection involved policy document review, male/female same-sex FGDs with health workers, national and district level key informant interviews (KIIs), and baseline documentation of health employees’ understanding of sexual harassment and policy guidance available at UMOH worksites. Phase 2 involved mixed-sex FGDs to elicit gender, social class, regional and ethnic dimensions and interaction and reporting dynamics, and in-depth interviews (IDIs) with employees or facility in-charges to deepen understandings of evidence that had emerged in Phase 1. See Additional file 3, for a detailed description of the methodology and sample.

Data collection took place between August–November 2017 with assistance from the (now-ended) USAID-funded, IntraHealth-led Strengthening Human Resources for Health (SHRH) project. The study sites included those of the UMOH and other central-level ministries, district human resources management (HRM) structures, hospitals and health facilities. The two categories of study population were key informants at the national and district levels and health workers and managers at health facilities. District-level data were collected in the ten districts in which the sexual harassment prevention and response system would be piloted. The districts were selected from the 44 project priority districts where there were staff shortages and where second year project efforts were concentrated. A purposive sample included 294 health workers (including managers) from Central (Mukono and Mubende); East Central (Bugiri, Namayingo); East (Tororo); Karamoja (Abim); North (Gulu); West Nile (Adjumani); West (Hoima); and South West (Rukungiri).

### Data collector training

Data collectors were trained to understand the protocol, tools and ethical requirements, and to address their attitudes about and experiences of sexual harassment, so that they would be at ease discussing issues that might initially might be met with reticence or discomfort, or might require probing. Female data collectors facilitated and recorded FGDs. Data collectors were also provided a five-day methodology training to reinforce their skills in collecting data using particular qualitative techniques (e.g., pile sorting), recording data and developing transcripts. Following training was a one-day pre-test and revision of the data collection tools.

### Data analysis

**Fig. 1:**
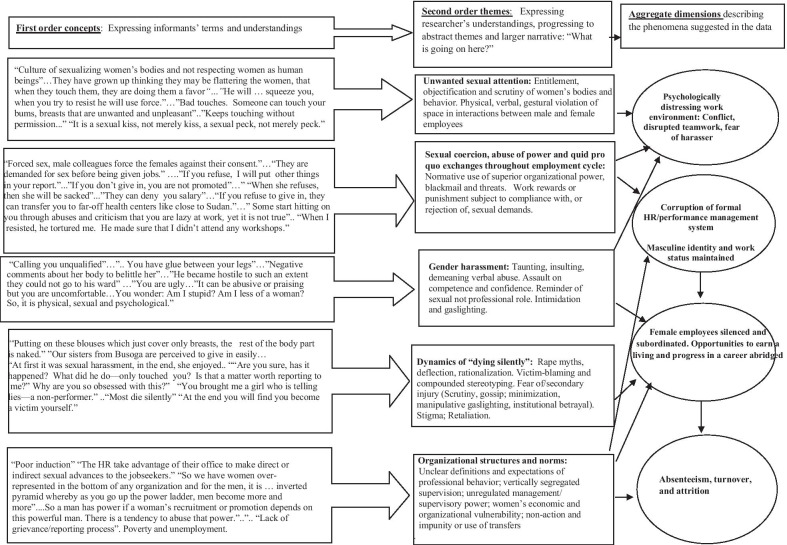
Employees’ experiences of sexual harassment, dying silently and consequences (Drawn from FGDs, IDIs, KIIs)

The transcripts were coded by research assistants who had participated in data collector training, and who had had previous experience with Nvivo. They were supervised by a research consultant with a background in anthropology and sociology. The research assistants read through the transcripts and became immersed in the data. The code structure evolved inductively. The researchers adapted the Gioia et al. “First-order/second order’’ analysis approach [69], and later created a data structure visual to graphically represent how analysis progressed from raw data to higher-level understandings and inter-relationships, and connections between the data and theory (see Fig. 1):

1. “First order concepts” expressed informants’ understandings (For example, “They are dressing indecently’);
2. “Second order themes” expressed researchers understandings, i.e., abstract-level concepts and themes and a larger narrative describing “What is going on here?” in theoretical terms (For example, “Indecent dressing’ suggests victim-blaming”);
3. Aggregate dimensions that might help explain various concepts and themes suggested by the data; and
4. Revisiting the relevant literature to see whether the research findings had precedents and if they had revealed new concepts.

## Results

Findings for this paper were synthesized from the transcripts and unpublished report text, and presented both in this section and in Additional files 4, 5, 6, 7. Additional file 4 illustrates the forms and examples of sexual harassment emerging from FGDs, IDIs and KIIs, including the physical, verbal, written/visual, and gestural behaviors and dynamics featured in health employees’ descriptions of sexual harassment. Additional file 5 contains excerpts from two key informant transcripts describing *secondary injury*, to illustrate the additional risks and harms faced by targets who had reported sexual harassment. The extensive use of quotes in the text below aims to convey health workers’ lived experiences or informants’ observations, and how they made sense of sexual harassment, in their own voices. Quotes were selected to illustrate categories and types of sexual harassment, its power dynamics, contributors and consequences. Findings in this section answer the two key questions: *What are UMOH employees’ experiences of sexual harassment? Why is non-reporting of sexual harassment pervasive?*

## Employees’ experiences of sexual harassment

There appear to be patterns of male-on-female aggression featuring sexual coercion and *quid pro quo*, unwanted sexual attention and gender harassment. Abuse of organizational power to coerce sex by managers and supervisors occurred throughout the employment cycle (see Table 1).

**Table 1:** Abuse of organizational power by managers and supervisors throughout the employment cycle

At **recruitment,** women are asked for sex and men are asked for money During **orientation**, the old staff may start to frequent your office and in the end, they may start to sexually harass you Delays in **confirmation** of your service—some people have taken long without being confirmed Delayed **promotion** even if you work for many years During **appraisals**—need a good report **Disciplinary action** can be taken selectively—if it is a lady, she may not be called for disciplinary action, but if it is a man, he will be called immediately for disciplinary action **Misuse of funds**, e.g., those who are heading certain facilities are given a basic fund for the staff, but you will find that they will be sharing it between the boss and the sex mate Sexual harassment comes as **orders.** So whether you are harassed or not, it comes as an ‘order.’ Use of authority to **schedule a duty** which is not necessary—from there, sexual harassment can occur Some others have power to **dismiss** because if you don’t give in, you are at a risk of losing your job	

Sexual coercion started during recruitment of health workers and continued after hiring, perpetrated by men in hierarchically superior decision-making positions– supervisors, senior managers (including human resources) or medical superintendents. Female applicants were promised or given jobs in exchange for sex. Such transactional behavior continued into the job where in-charges/supervisors offered bribes and rewards, such as gifts, exemption from night duty, working fewer hours, and opportunities for training or promotion, in exchange for sex**.** Refusal was followed by further coercion, reprisals or psychological or administrative retaliation.

The following quotes express experiences and observations of sexual harassment in UMOH workplaces.

### The abuse of superior organizational power

“I have always received reports about sexual harassment and it has taken different forms. Usually it takes the form of power relations when people are looking for jobs in the public service. The HR feels they have more power and they take advantage of their office to make direct or indirect sexual advances to the jobseekers. For example, they could tell the jobseeker that there is no job available and that the jobseeker should keep coming back to the office or that there is no free work, or they could ask the jobseeker to come to the office at awkward hours.” (Female National-level Key Informant).

“So we have women over-represented in the bottom of any organization and for the men, it is an upward or inverted pyramid whereby as you go up the power ladder, men become more and more and are fewer at the bottom….so a man has power if a woman’s recruitment or promotion depends on this powerful man. There is a tendency to abuse that power and they don’t even think that they are abusing it because they have grown up thinking they may be flattering the women, that when they touch them, they are doing them a favor. So, it is the whole culture of sexualizing women’s bodies and not respecting women as human beings. Their dignity, not looking at them as individuals who have bodily integrity and a choice. When you combine all those, it is really what causes sexual harassment.” (Female National-level Key Informant).

### Sexual coercion and quid pro quo (Female health worker FGDs).

*“Demands for sexual favors are common for ladies. They are demanded for sex before being given jobs.*”

“*Supervisors demand for sex from females in offices with threats of sacking upon refusal.*”

“*They wait for you at the time of appraisal and harass you*.”

*“Most of the sexual harassment occurs to the trainees in the health sector who are coming for their practice. Most trainees will try to look for a place where they can do their practice. But there is no place and the only place they find, someone tells them that if you do not sleep with me, you do not get the opportunity. So somehow you have to weigh between the two, which one is better: Should I sleep with him and get the opportunity or I leave?”*

“*Forced sex, male colleagues force the females against their consent… The boss can call you in his office and force you to have sex with him*.”

*“But sometimes it is economical because you have refused to give in to the sexual favors, you are denied some economic benefits- it could be allowances, field trip, salary reduction, transfer so that your economic chances are reduced depending on how you give in to the sexual favors or not. …There are people who rotate in workshops or trips. If it is field, she is the one, workshops- she is the one in charge of the money/accounts, she/he is on every list…so you are denied of some economic benefits as a way of harassing you to go into a sexual demand.”*

*“Some start hitting on you through abuses and criticism that you are lazy at work, yet it is not true…when you give in the abuses stop”.* Also, see Table 2.

**Table 2:** A Health Worker’s Experience of Non-Consensual Touching, Coercion and Psychological Distress [In-Depth Interview]

**I: Can you please describe sexually harassing behaviors you are acquainted with?** **HW**: In my former workplace, I had a male in-charge. I was pregnant at the time. This guy wanted to go in a deep relationship with me. I had heard that he would try to create a relationship with whoever was pregnant in that department. And if the person accepted, he would be in a deep relationship with her. I underwent serious struggles during that time because I had just joined the system. This guy *tortured* me. In my third trimester, he said to me, “you are here and pregnant- you have come to work for a few months and you will go for your maternity leave, now what do you want? It was better for you to first deliver your baby before starting work.” I was clueless on what action to take because I was a contract worker under the MOH. But what hurt me most was that this guy wanted me in the TB ward despite my low immunity as a pregnant woman. He wanted me to get exposed to so many things. Yes, I had to work, but there are some occupational hazards you are exposed to in the workplace. Another challenge was that after delivering my baby, this guy wanted me back at work before the end of my maternity leave because I had combined maternity leave with annual leave. He wanted me to work and did not want to give me time off to care for my baby. I was very weak. I couldn’t stand for so long and I couldn’t sit for so long **I: Had he ever proposed to you?** **HW**: Yes, he did. Actually, the way he could harass me like he would come and touch me **I: Touch where?** **HW**: He would touch my bum. When I resisted, he *tortured* me. He made sure that I didn’t attend any workshops. Yet colleagues, who started the job at the same time as me, went for workshops and outreaches. Whenever I requested to attend an outreach in order to gain some knowledge, he would say, “No, you cannot go.” But my pregnant coworkers and coworkers with new-born babies attended the workshops	

### Unwanted sexual attention

Findings in Additional file 4 illustrate the objectification and sexualization of female health workers, non-consensual touching and gestural and verbal behaviors that violate the space of the target through unwanted, unwelcome attention.

#### Non-consensual touching (Female health worker FGDs)

*“Bad touches. Someone can touch your bums, breasts, and some other parts that are unwanted and unpleasant. Someone can come and touch on your nose and chin.*“

“*Touching the person between colleagues, for example the male touching the breasts of the female when she doesn’t want it-they are just working together-but this person keeps touching without permission.”*

“When greeting some men, they can tickle/scratch inside the female’s hand.”

“It is a sexual kiss, not merely kiss, a sexual peck, not merely peck.”

### Gender harassment

Evidence of “gender harassment” emerged from FGDs. This category of harassment consisted of bullying, taunting, derogatory, sexually gross, insulting verbal abuse by co-workers and supervisors, including the use of threats or acts of retaliation. Targets apparently did not take the behaviors in Table 3 as sexual invitation**.**

**Table 3:** Examples of Gender Harassment with Unwanted Sexual Advances and Reprisals [From Female Health Worker FGDs]

*“Provoking you. Calling you unqualified when you refuse sexual advances… So, words spoken- telling you how you are sexual, how you are not using your endowment, how you are not exploiting yourself for higher offices, abusing you, you are ugly that’s why you have nobody loving you.”* *“You are told how beautiful you are. Like that name calling- sweetheart, honey, Virgin Mary- an older woman wonders why someone calls her a Virgin Mary, because we have not seen you sleep around! You are told how you are “magulu gaamu” (meaning have glue between your legs, can’t give in for sex)—we wonder who sleeps with you, you refuse giving us.”* *“And also making comments about the lady—he makes a pass at the lady, but she responds in a negative way, so he starts making negative comments about her body to belittle her, to annoy her.”* *And intimidating words, someone comes and tells you …you think your husband is faithful to you?”* *“Using vulgar words which are not good for our mouth to pronounce.”* *“He became hostile to such an extent that they could not go to his ward.”*	

Together, the female health worker FGDs provide a narrative in which the experience of harassment involved psychological manipulation (*gaslighting)* which resulted in confusion, loss of confidence and psychological distress:

*“The approach can be different- can be abusive or praising but you are uncomfortable with the comment... It is psychological… In your mind you wonder- Am I stupid? Am I less of a woman? So, it is physical, sexual, and psychological.”*

*“You are ugly--‘Whom do you think is interested in you?’ when he was actually interested in her.”*

“Somebody will be psychologically tortured like what happened to the other student nurse. This is PTSD-Post Traumatic Stress Disorder.”

### Patients as targets of sexual harassment

Evidence of the sexual harassment of patients by clinicians emerged unexpectedly from the Phase 1 health worker FGDs. This was followed-up by interviews with 16 facility In-Charges in the facilities where this behavior was reported. Irrelevant or unnecessary vaginal and breast exams and “bad touching” were reported to be the most common forms of sexual harassment by clinicians, though forms also included displaying a patient’s nude body or body parts during clinical exams, and sexual assault such as rape. Facility In-Charges observed that victims rarely reported the health worker to police, hospital administrators or others, likely for fear of negative consequences. For example, a Facility In-Charge recounted a story in which a young woman who had been sexually assaulted by a clinician complained to her family and the village chief, and the latter asked her not to report it further lest the village lose the one health provider they had or possibly receive less preferential treatment in the future [see Additional file 7, Patient Harassment by Health Workers].

When asked about contributed to sexual harassment in general, male FGD participants mentioned the following examples which are relevant to the harassment of patients: Medical examinations and clinical procedures make clinicians vulnerable to harassing, such as palpation of a female client, collection of vaginal swabs, making an injection on the thigh of a female patient or observing a female worker insert a catheter in a male patient. This finding suggests that responsibility for harassing behavior is *deflected* onto a medical task or onto a female body which during examination has aroused the perpetrator.

### Contributors/causes of employee harassment

Study respondents identified contributors to sexual harassment at several levels.

### Individual and relationship-level contributors

A recurrent theme in focus groups was a belief that women’s “indecent dressing” caused sexual harassment, *deflecting* attention onto the target who was said to be responsible for calling forth harassing behavior. Stereotypical notions seemed to portray men as victims of seductresses whose manner of dress or walking enticed perpetrators:

*“Dressing still on ladies, like putting on these blouses which just cover only breasts, the rest of the body part is naked. There is this term they use to call this type of dressing, pimp dressing. You see they put on this thing up to here; they call it cleavage dressing (the navel just remains outside (The kundi shows*).” (Male Health Worker FGD)

*“One of the other causes, I think is dress code. Because if a lady dresses up and the dress is revealing most of her body parts, that entices the opposite, to initiate and at the end of the day, call it sexual harassment.”* (Male District-level Key Informant)

*“…One thing I would really point out is that somebody may be doing something subconsciously and it can be construed as sexual harassment. For example, if a lady was walking with her high-heeled shoes and she is wriggling in the corridor while there are other onlookers, that can’t be harassment...”* (Male District-Level Key Informant) (Table 4)

**Table 4:** Perceived Contributors to Sexual Harassment (Health worker and managers FGDs and KIIs)

*Perceived individual- or relationship-level contributors (from FGDs and KIIs)*	
“Indecent dressing”—women’s clothing provokes it Provocative walking– women’s behavior provokes it Medical examinations—women’s state of undress provokes it Living away from spouse Proximity to co-worker Alcoholism Libido Morals/poor upbringing	
*Perceived organizational contributors (from FGDs and KIIs)*	
Abuse of power Manager/supervisor power Unclear expectations of professional behavior Poor induction of new hires Unclear definitions Lack of grievance/reporting process Lack of privacy in sleeping quarters Belief women can be touched Impunity for touching, sexual harassment Only sexual assault or rape is taken seriously	
*Perceived cultural/societal contributors (from FGDs and KIIs)*	
Poverty and unemployment Regional, ethnic and gender traits Non-consensual touching of women	

In the following job-seeking scenario, persistent, coercive sexual demands in an imbalanced power relationship are described as normal courtship behavior, in the face of the target’s attempts at resistance:

“At first it can be harassment but later, it becomes enjoyable. Let us take this scenario of these ladies who are used when they want jobs…There is a lady I know who wanted a job and the boss demanded for sex and the lady gave in. After giving in four times, the boss ended up marrying the lady…so it started as sexual harassment, later on it was not. This lady initially refused saying she is born again but after giving in, she accepted and enjoyed the marriage.” (Male Health Worker FGD).

Organizational factors, including inaction and impunity, emerged as major contributors to sexual harassment:

“I think the most common cause is that in Uganda, there are no punitive actions against sexual harassment. You know you can touch a woman and get away with it. You cannot report to police that so-and- so touched me since even the police are some of the main perpetrators…When you report to the local council they will not help you because they think it is normal for women to be touched. The only thing they can listen to is rape.” (Female Health Worker FGD).

“There was one case scenario—this was not an employee but then it was someone seeking for a service in one of the public places, and it happened to be a nun. She went to this person and this person without shame turned to this person and started demanding for sexual favors from the nun. Of course, nun made an alarm that attracted attention and probably the church should have started from there but…no decision was taken.” (National-level Key Informant).

### Beliefs about regional, ethnic and gender attributes

Additional file 6 presents FGD and IDI data on perceptions of regional and ethnic attributes that intersect gender beliefs and that explain sexual harassment. For example, women or men from urban regions were stereotyped as “easy” (e.g., “Dot.com Girls”). Traits attributed to Banyakole, Busoga or Batooro women and men were also implicated in perceptions of sexual harassment.

### (Why) is non-reporting of sexual harassment pervasive?

This section presents data on reactions to and consequences of sexual harassment as well as silencing dynamics in UMOH workplaces that shed light on non-reporting.

### Reactions to sexual harassment

FGD participants were about asked common reactions to sexual harassment. Table 5 shows that transfer/ absconding (leaving the job) tied with avoiding/resisting/ignoring the harasser as the most common reactions.

**Table 5:** Reactions of Health Workers (From FGDs and IDIs)

**Reactions**	**No. of times mentioned (More than one time in 26 FGDs)**	**No. of times mentioned (10 IDIs)**
Leave the job (Transfer/abscond)	15 (58%)	
Ignore, resist, avoid harasser	15 (58%)	8 (80%)
Comply, give in	12 (46%)	5 (50%)
Talk to friends, colleagues	7 (27%)	
Quarrel with harasser	12 (46%)	
Report the incident	13 (50.0%)	3 (30%)
Keep quiet		2 (20%)

These common reactions and compliance with sexual demands suggest silence or non-reporting. However, quarreling and reporting (46%, 50%) indicate that reactions other than silence exist, i.e., that sexual harassment is resisted and reported to someone. It should be noted, however, that no formal UMOH reporting system existed at the time.

### Perceived consequences

Fear of the consequences of sexual harassment which involve retaliation suppresses reporting. See Table 6, items 12–17.

**Table 6:** Personal and professional consequences of sexual harassment (Female health worker FGDs**)**

1. Loss of self-esteem/dignity 2. Loss of interest in the work 3. Lower productivity 4. Psychologically affected: Feeling stigmatized, depressed, guilty, self-blame, trauma 5. Conflict with spouse/divorce 6. Drop in work performance due to stress 7. Decreased job satisfaction 8. Absenting oneself from work/absconding 9. Health consequences: HIV/STI, unwanted pregnancy, abortions 10. Relationship between perpetrator and target undermined 11. Poor work conditions 12. Bad comment on your performance appraisal (Retaliation) 13. Delay in your confirmation (Retaliation) 14. Deleting your name from the payroll and you miss your salary (Retaliation) 15. Demotion when you refuse to give in (e.g. supervisor can demote you from being an in-charge of a ward to a mere nurse (Retaliation) 16. Loss of job, promotion or economic benefit (Retaliation) 17. Unwanted, punitive transfers (Retaliation)	

Actual loss of employment was a consequence, as “*Sometimes you can even lose your job. The man may make advances at you and you always say no and at times may decide to leave the job. I have a friend who left her job and went to sit home because the boss was always demanding her for sex which she could not accept.”* (Female Heath Worker FGD). Punitive transfer can occur “*If you refuse to give in to sexual harassment… they can transfer you to far-off health centers like close to Sudan.”* (Female Health Worker FGD). In addition to personal and professional consequences, study respondents mentioned the effects of sexual harassment on work climate, including: Conflict and disrupted teamwork; fear of or loss of respect for the harasser; undermining the relationship between harasser and target; and undermining of supervisory authority.

### Dynamics of silencing

The two cases described by national-level stakeholders in Additional file 5 illustrate the *secondary injury* or revictimization that may result from reporting and that silences reporting. In Case 1, retaliation, excusing the harasser and attempts at normalizing sexual harassment as an expected part of life, are described. Case 2 illustrates the disbelief and minimization involved in manipulative gaslighting, where the hierarchical superior appeared to challenge the target who attempted to report: *“Are you sure, has it happened? What did he do—only touched you…? Is that a matter worth reporting to me?” “Why are you so obsessed with this?* Also, there is damage to reputation and a countercharge of false allegation: “*You brought me a girl who is telling lies—a non-performer.”* In the second case, the target is described as losing her educational program, her job and her marriage.

A national-level key informant described *institutional betrayal* in this way: *“In Uganda, reporting is quite low…most people fear to report because of the consequences—you don’t know what will end. At the end you will find you become a victim yourself. You think you are trying to salvage yourself from the challenges you are facing by using the channels available to talk about your boss – who is harassing you sexually, but instead, it will turn against you.”*

Feelings of shame, embarrassment, and fear of exposure, gossip and stigma hindered reporting. A target who considers reporting fears heightened scrutiny or publicity, gossip, disbelief, intimidation or cover up:

*“Some victims do not want the issue to be said out. They feel ashamed when the community gets to know they were sexually harassed. So, when the witness tries to bring it out, denial is also there. When the victim denies, it makes it difficult for the witness to proceed with the case. Sometimes the harasser may be on side of you both, so they will just sit on your issue…”* (Female Health Worker FGD)

*“When you are a victim and you report, you become the talk of the city. It is not that whenever you report all people will believe you. You may report and people think you are deceiving. So, it is better you keep quiet.”* (Female Health Worker FGD)

*“You may talk to a friend of yours seeking advice, that so- and- so wants to give you a job but you first have to give him sex. That friend will ask you, do you remember the number of years you have spent without a job? Just give in, the secret will be between you and the boss. And you will remain with ‘your thing’ and life continues. People no longer take it seriously.”* (Female health worker FGD)

*“I was at the pediatric ward and so I used to take blood sample to the lab. I think that is where he saw me from and he started picking interest in. He started talking to me and finally, he told me what he wanted. I personally told him that I cannot be involved in such relationships because I am married. The whole time I worked at the hospital, I never told anyone until when I was leaving for more studies. He was ever on my neck and yet he is even an old man.”* (Female IDI)

*“Intimidation from the boss and the boss tells you even if you report no one will support you. ‘We are known’ and you end up keeping quiet.”* (Female Health Worker FGD)

The findings demonstrate the feared and real risks of secondary harm and institutional betrayal related to reporting. As a national HRH key informant observed: “*It is a complex thing that most people die silently. Like for the common cadres, they will ask for a transfer to other facilities away. Most of the people suspected to be harassing their subordinates, especially sexual harassment—at best what they do is also transfer them to other places and there are quite a number of scenarios, so what they do is to transfer the person to other facilities or another department. So, because of that, most people don’t see the reason to go and report and say [they] would rather die with the problem.”*

### How the UMOH took the study results into account

*“*Dying silently” renders sex-based harassment formally invisible. The research results were disseminated within the UMOH and among stakeholders, were used in 2018 to develop gender-transformative *Guidelines to Implement the Policy on Prevention and Response to Sexual Harassment,* and posted on the UMOH website later in 2018 [70]. The UMOH *Guidelines* used research evidence to create several entry points for speaking about harassment, and to shift understandings of sexual harassment. For example, The *Guidelines* used the study participants’ own experiences of sexual harassment to illustrate its forms, thus *shifting the power* to define sexual harassment to the target of harassment, and allowing the target to “reclaim the narrative” [71]. The UMOH *Guidelines* also provided policy directives to health sector employees related to two particular abuses documented by the study: Supervisor/supervisee relationships and sexual harassment by clinicians of patients. These policy “guardrails” aimed to transform aspects of gender power relations. In 2021, a workplace climate improvement survey will be conducted in 30 health districts of Uganda’s Eastern region to improve health facility workforce governance capacity. See Additional file 8 for details.

## Discussion

In this section, we discuss the cross-cultural relevance of current theories; how sex-based harassment corrupts management/supervision systems (something overlooked in technical discussions of support supervision and performance management); power and gender inequality; the mutually reinforcing intersections of sex-based harassment and vertical occupational segregation; the harassment and abuse of health system patients and employees; and implications for HRH/M policy.

Figure 1 brings together the research results in a visualized data structure [68] to answer the questions, *What are UMOH employees’ experiences of sexual harassment? Why is non-reporting of sexual harassment pervasive*? First-order concepts expressed informants’ experience of sexual harassment in their own words. Second-order themes suggested three categories of *sex-based harassment,* the dynamics of non-reporting (“dying silently”) and aspects of organizational structures and norms that perpetuate sexual harassment. Figure 1 also depicts aggregate dimensions, i.e., the broader HRH effects such as a psychologically distressing work environment, the corruption of the formal performance and HR management/supervisory systems, female health employees’ subordination and abridgement of employment opportunity, and organizational consequences such as absenteeism, attrition and turnover (through requested or punitive transfers). The inter-relationships depicted between second-order themes and aggregate dimensions are not exhaustive.

The cross-cultural portability of organizational silencing dynamics, such as victim-blaming, retaliation, minimizing, deflection, gaslighting and institutional betrayal, is borne out in the study results [16, 61, 62, 64, 66]. Some of the theories of sexual harassment mentioned earlier also appear portable to UMOH workplaces. For example, sexual harassment as a natural extension of mate selection emerged in describing the targeting of a resistant job seeker with persistent sexual harassment as courtship and that the target eventually enjoyed it. Sex role/sociocultural “spillover” appears in the intrusion of pervasive, non-consensual touching in UMOH workplaces (“bayeye”). One could argue that economic motives underlay the punitive transfers to “*far-off health centers like close to Sudan,”* effectively kept female health workers from competing for valued and influential jobs traditionally held by men [14, 15]

The cross-cultural portability of *power-based theories* and the three categories of “sex-based harassment” were also demonstrated in study results. For example, unwanted sexual attention was embodied in persistent demands and non-consensual touching in frequent violations of women’s personal and professional spaces [16]. Sexualizing talk served to remind female health workers of their sexual, rather than professional, role (“*Words spoken telling you that you are not using your endowment”*). Superior and apparently unregulated organizational (management and supervisory) power was widely (ab)used to coerce sexual quid pro quos. As Hearn and Parker suggest, “nor is sexual harassment only a process of subordination and re-subordination of women as workers in a hierarchy. It has to be seen an individual appropriation of women, a male sex right” [16, p.13–14]. Sexual harassment functions as an agent of social control [7], with the practice of using women’s sexuality to keep women in subordinate positions as key to the way women are treated [6], at least in male-dominated workplaces. That gender inequality drives sexual harassment [26] is evident in the study data.

Gender harassment involved “gendered opprobrium” [34] such as *“abusive or praising but you are uncomfortable with the comment… In your mind you wonder- Am I stupid? Am I less of a woman?”* Female employees also described the denigration of being labeled “ugly” in response to refusal of unwanted sexual attention and coercion. Gender harassment has been described as not aimed at sexual cooperation, “more put down than come on” [33], though this research revealed Uganda-specific instances of gender harassment that seemed both “come on” *and “*put down” [20] in the service of unwanted sexual attention and coercion. However, consistent with prior descriptions, gender harassment did appear used to police appropriate ways of “doing gender” [23, 27], i.e., to enforce a feminine gender ideal of compliance that, when advances were resisted or rebuffed, ended in punishment. As a jurist once observed, it is “demeaning and disconcerting” for a worker to “run a gauntlet of sexual abuse in return for the privilege of being allowed to work and make a living” [72].

Cockburn’s [21] description of sexual harassment as an expression of power, not of unbridled desire, is an apt recapitulation of and prelude to understanding manager/supervisor harassment in the Uganda context:

“Hierarchies are expressions of differential power, maps of the distribution of authority and subordination in an organization. Men’s treatment of junior women (being touched, women-objectifying talk) is a clear instance of the exercise of sexual power … We see sexual harassment as being a male intervention for the assertion of power, as a warning to a woman for stepping out of her proper place. It is a controlling gesture to diminish any sense of power she may be acquiring and to remind her “you’re only a woman, that’s the way I see you. And at that level, you’re vulnerable to me or any man” [p.142].

In UMOH workplaces, managers and supervisors were structurally situated to exercise power and to control work-related outcomes [18] such as positive performance evaluations, promotions and opportunity for in-service training. Sexual coercion and blackmail emerged as a workplace pattern where men in higher status positions abused unregulated organizational power to intimidate and subordinate female UMOH employees, and extort sex, throughout the employment cycle (NB: There was only one mention of senior-level female-on-male sexual coercion, which was an “outlier”). Rewards and sanctions were levied in these seemingly informal “management” systems where compliance with sexual demands or suffering the penalties were “rules of the game” and where female health employees could expect little professional advancement without being expected to pay for it in sexual “currency” [73]. This suggests that the relationships, rewards and sanctions of formal HR management and supervision systems were corrupted by the abuse of unregulated organizational power. Kabatt-Farr and Crumley remind us that it is the hierarchy of health care that normalizes the power differentials between men and women that enables the harassment that protects superior status and excludes people from full participation in the workforce [74].

Transferring the perpetrator (“pass the harasser” [48]) or the target of harassment are both administrative dysfunctions in this informal performance management system. Supervisor-supervisee relationships and functions likely lost credibility through routine attempts to coerce sex*.* It seems reasonable to suppose that these processes directly or indirectly contributed to absenteeism, turnover and attrition—the very workforce shortages that dogged the health sector and that should have been addressed by HRM systems. Technical discussions of performance management and supervision appear to overlook these possible systems’ corruptions.

It has been argued that sexual harassment is not meant to appeal to women—it is meant to coerce them [75]. When the target has no choice in the encounter, or has reason to fear the repercussions of refusal, the interaction has moved out of the realm of invitation, courtship or flirtation, and into the realm of intimidation and aggression [75]. Schultz suggests that, once *sex-based harassment* is understood as a means of protecting hegemonic masculine work status and identity, “even unwanted sexualized attention becomes visible as a means of putting women down” [34], i.e., of maintaining subordination. The UMOH results also evoke the ongoing difficulties for female employees to progress beyond sexual objectification and subordination and to establish professional credibility under conditions of poorly regulated sexual aggression. These harassing processes not only undermined the HRM goal of retention, but also abridged opportunities for female employees to engage in economically productive work and progress in a career. The mutually reinforcing intersections of sexual harassment and vertical occupational segregation appear to be related discriminations and not independent obstacles experienced by women seeking leadership positions.

Psychological and physical violence and organizational violations [16] in the forms of sexual coercion and blackmail, gender harassment, the dynamics of silencing, retaliation and secondary injury appeared so pervasive in UMOH workplaces as to suggest organizational norms and systems. For example, an abuse of power and the corruption of management/supervisory appear normative. The abuse of superior power is also apparent in the “sextortionary” [73] harassment of female clients by clinicians and also suggests normative organizational processes. Such breaches of trust by persons who abuse the social power derived from their positions, and the silence surrounding these processes appear *systemic* and have relevance for the conceptualizations health system governance, HRM and the quality of services.

### Implications for HRH/M Policy

The study results have implications for definitions and measurement, prevention and response. First, sexual/sex-based harassment presents as a systems corruption, not a localized problem. Second, that sex-based harassment is a manifestation of gender inequality. The term “sexual harassment” fails to address the unequal gender-relational and “put-down” behaviors captured by the category of *gender harassment* in “sex-based harassment.” Three categories may better account for the range of violence and discrimination that female health workers may face at work.

Given the foregoing, what interventions and mechanisms are most likely to be effective in prevention and response? The first answer is this: Policies and interventions should target gender inequality and link efforts to end workplace violence and harassment with efforts to end other forms of inequality and violence in the health sector [26]. Moreover, since sexual harassment involves individuals, groups, communities (including organizational “communities") institutions and structures, policies and interventions should be multi-level and follow an ecological model [26] similar to other gender-based violence prevention and response efforts.

The second answer is aimed at HRH/M leaders: Interventions should target organizational change. Research has demonstrated that the most powerful determinant of sexual harassment is organizational climate and its tolerance of harassment [74, 76]. Changing an organization’s climate may involve leadership dissemination of a zero-tolerance policy, management visibly taking all complaints seriously, reporting on the results of investigations and following through on sanctions [74] to end impunity. The problem of sex-based harassment should be “spoken” [16] through reporting systems that are safe and not punishing to targets. However, there is not much evidence that internal complaint processes, alone or as they are currently designed, prevent sex-based harassment, because typically leave in place the broader organizational drivers that perpetuate harassment [24], rely or focus on individuals and expose the individual target to professional, economic and psychological risks. Certainly, formal and non-formal grievance mechanisms should be available to employees, but take note of a 2002 study that concluded that it is *unreasonable* to report sexual harassment, and that sometimes the most reasonable course of action is to avoid reporting, when the organizational response is likely to minimize the experience, where there are procedural difficulties, or when a lack of leadership commitment contributes to greater negative and psychologically distressing effects [76]. Kabatt-Farr and Crumley point out that targets of harassment develop multiple coping strategies that may or may not include reporting and that it is unfair and uninformed to disregard the experience of harassment simply because it is not reported [74]. Wear and Altman recommend that the focus of inquiry should be on the institutional environment and not on the target’s report [67]. Efforts to eradicate an ongoing culture of workplace of sex-based harassment must avoid policies or practices that suppress reporting: Standards that make sexual harassment actionable only if cases are “severe” and “pervasive,” nondisclosure agreements or forced mediation/arbitration [77], as well as anti-retaliation policies that fail to anticipate the “dynamics of violation” and the pressure to silence complaints once they are “spoken” [16]. Testing additions or alternatives to individual, face-to-face reporting include workplace climate surveys or an *information escrow* system that would allow a target to place a private complaint into the custody of a third party, to take effect only when a specified condition has been fulfilled, e.g., a complaint is lodged with authorities if an “escrow agent” receives at least one additional allegation of sexual harassment against the same individual [78].

Training employees by itself, and as it is typically designed, has likewise proven ineffective [79]. A bystander program, which takes the onus of ending sexual harassment off the shoulders of the individual and places responses on the organizational community, targets changes in behavioral/ organizational norms and may be effective if bystander training integrates context-specific research evidence on victim-blaming and other silencing dynamics, and links sex-based harassment to sexism, gender inequality and other forms of gendered violence[26], in addition to training witnesses to disrupt harassing behaviors in safe ways [80, 81]. It is likely that the prevention of violence and harassment would be more effective if bystander programs were established upstream during heath professional training [82].

Researcher-practitioners have already identified a key (gender-transformational) structural intervention: “*We already know how to reduce sexual harassment at work, and the answer is pretty simple: Hire and promote more women”* [24, 79]. This may be difficult in the physician-dominated healthcare setting [74], yet real gender parity will likely provide the authority, strength and safety to counter stereotypes, resist harassment and contribute to reshaping non-sexist organizational norms and cultures [24]. Flattening the hierarchy [74] may ultimately be necessary to regulate unchecked, subjective and arbitrary HRM/supervisory authority [24], though preventive action can be taken during hiring, induction and ongoing employment and management processes, such as outlining the parameters of management/supervisory authority, communicating examples of abuses of management power and conflicts of interest backed up by a written professional code of conduct, clearly defined consequences for infractions and due diligence follow-up.

The practice of transferring a serial harasser (“pass the harasser” [48]) must be named as an HRM and health system dysfunction that contributes to impunity. “Sextortion” by supervisor or clinician brings into sharp relief the need for sectoral as well as organizational intervention to disrupt systemic abuses of power and authority. Effectively ending sex-based harassment may require anti-corruption measures that have been effective in other sectors [73, 83, 89–91] including: The use of an independent external reporting and investigation mechanism; linking to other anti-corruption or good governance efforts; collective action with gender justice groups; guidelines, sanctions and disciplinary measures against institutions and individuals found to have perpetrated corrupt/sextortionary practices; and professional codes of conduct. In Uganda, in the UMOH worked with health professional councils to integrate zero tolerance in professional codes of conduct [see Additional file 8].

Good workforce governance and management require a gender-aware, human rights-based approach that holds employers accountable through HRM policies and practices which reflect international human rights and labor standards that protect health workers from gender-based harassment, violence and discrimination in the workplace [40, 42, 84, 85]. It has been observed that Human Resource departments function to protect the organization, not the employee [86]. In contrast, human rights-based HR would facilitate access to justice, through administrative measures to end impunity as well as access to legal remedies where administrative measures prove ineffective [85, 87].

Research on health system dysfunction should survey and make visible incidents of sex-based harassment in efforts to create health workplaces that offer “decent work” [88] to employees and high-quality services to clients. Future research should assess often-hidden patterns of sexual/sex-based harassment, including organizational context- and culture-specific patterns of coercion, gender harassment and unwanted sexual attention and leadership tolerance of these; the dynamics of sex-based and racial/ethnic and other culturally-relevant bases of harassment, the prevalence of “sextortion” of both patients and employees, including their (similar or different) dynamics, effects and consequences, and test administrative and legal measures that effectively disrupt them. Health system strengthening in the time of COVID-19 suggests that research should track how this social disruption may exacerbate or disrupt, or create new, patterns of sex-based abuse and vulnerability in health systems.

## Conclusions

Sex-based harassment created distressing work environments in UMOH workplaces, abridged female health workers’ rights and opportunities and patients’ safety, corrupted HRM and performance management systems and undermined the achievement of human resource systems’ goals. Health systems leaders should seek organizational and sectoral solutions to end sex-based harassment and make gender equality and the protection of employees’ rights HRH policy priorities.

## Supplementary Information

**Additional file 1: **Illustrative Definitions.

**Additional file 2:** Definition and Interpretation from Uganda’s 2012 Employment (Sexual Harassment) Regulations.

**Additional file 3:** Data Collection Methods and Sample.

**Additional file 4:** Forms and Examples of Sexual Harassment Experienced by Health Employees.

**Additional file 5:** Two Cases of Secondary Injury after Reporting Sexual Harassment.

**Additional file 6:** Regional, Ethnic and Gender Stereotypes.

**Additional file 7**: Patient Harassment by Health Workers.

**Additional file 8:** How the MOH Took the Study Results into Account.

## Data Availability

Datasets and transcripts were destroyed after the study was completed as per the protocol. Data necessary to interpret, replicate and build upon the findings are in unpublished text files available from the corresponding author on reasonable request.

## Abbreviations

GBV: Gender-Based Violence
GDIA: Gender Discrimination and Inequality Analysis
HR: Human Resources
HRH/M: Human Resources for Health/Management
HSS: Health Systems Strengthening
KII: Key Informant Interview
IDI: In-Depth Interview
FGD: Focus Group Discussion
UMOH: Uganda Ministry of Health

## References

[1] UN Women. Towards an end to sexual harassment: the urgency and nature of change in the era of #MeToo. 2018. p. 11

[2] European Union Agency for fundamental rights. Violence against women: an EU survey. Main results. 2014.p. 95–96. https://fra.europa.eu/en/publication/2014/violence-against-women-eu-wide-survey-main-results-report

[3] #MeToo Impact Report. 2019. https://metoomvmt.org/wp-content/uploads/2020/01/2019-12-09_MeToo_ImpactReport_VIEW_4.pdf2020/01/2019-12-09_MeToo_ImpactReport_VIEW_4.pdf. Accessed Oct 20, 2018

[4] Uganda Ministry of Health Workforce Study (2003). Satisfaction and intent to stay among current health workers.

[5] Newman C, Mugisha M, Matsiko C. Uganda Ministry of Health gender discrimination and inequality analysis report. IntraHealth International, Chapel Hill. 2012. https://www.intrahealth.org/sites/ihweb/files/files/media/uganda-ministry-of-health-gender-discrimination-and-inequality-analysis-report/GDIA-Report.pdf

[6] Tamale S (1999). When hens begin to crow: Gender and parliamentary politics in Uganda.

[7] Kyomukama S. Sexual harassment at the workplace: A case study of the Uganda police force. 2004.

[8] Aloka J. Prevalence and management of sexual harassment at the workplace: The case of Uganda prison service (2002–2006). A thesis submitted in partial fulfilment of the requirements for the award of the degree of Masters of Arts in public administration and management of Makerere University. 2009

[9] Research Triangle Institute and World Vision. School-related gender-based violence formative assessment. USAID/Uganda Literacy Achievement and Retention Activity. 2018

[10] The Guardian. 'Men fear us': Kampala's market women unite against harassment.” Nd. https://www.theguardian.com/global-development/2019/aug/19/men-fear-us-kampalas-market-women-unite-against-harassment

[11] Attributed to Albert Einstein. Not everything that counts can be counted, and not everything that can be counted counts. http://www.braintrainingtools.org/skills/not-everything-that-counts-can-be-counted-by-albert-einstein/10.1177/2049463714565569 PMC4616986 26516551

[12] Pina, A, Gannon TA & Saunders, B. An overview of the literature on sexual harassment: Perpetrator, theory, and treatment issues. Aggression and Violent Behavior (14). 2009. 126–138. P. 130.

[13] Advocates for Human Rights. Causes, theories and effects of sexual harassment. http://www.stopvaw.org/Theories_of_Sexual_Harassment. Accessed February 2020.

[14] Yount KR (1991). Ladies, flirts and tomboys: strategies for managing sexual harassment in an underground coal mine. J Contemp Ethnogr.

[15] Friedman GS, Whitman JQ. The European transformation of harassment law: Discrimination versus dignity. SSRN Journal. 2003.

[16] Hearn, J, Parkin. Gender, sexuality and violence in organizations: the unspoken forces of organizational violations. 2001. Sage Publications.

[17] Connell R. Gender, health and theory: Conceptualizing the issue, in local and world perspective. *Social Science and Medicine.* (2011) pp. 1675–1683. 10.1016/j.socscimed.2011.06.006 21764489

[18] O’Connell CE, Korabik K (2000). Sexual harassment: The relationship of personal vulnerability, work context, perpetrator status, and type of harassment to outcomes. J Vocat Behav.

[19] World Health Organization. Delivered by women, led by men: A gender and equity analysis of the global health and social workforce. Human Resources for Health Observer Series No. 24. 2019. https://www.who.int/hrh/resources/health-observer24/en/

[20] Advocates for Human Rights. http://www.stopvaw.org/Violence_and_Power; http://www.stopvaw.org/Perpetuation_of_Gender_Stereotypes; http://www.stopvaw.org/Economic_Power_over_Women. Accessed Feb 2020.

[21] Cockburn C. In the way of women. Men’s resistance to sex equality in organizations. Cornell International Industrial and Labor Relations Project. ILR Press: Ithaca, NY. 1993. Number18. 141–142

[22] McKinnon C (1979). Sexual harassment of working women: a case of sex discrimination.

[23] Konik J, Cortina L (2008). Policing gender at work: intersections of harassment based on sex and sexuality. Social Justice Res.

[24] Schultz V. Open statement on sexual harassment from employment discrimination law scholars. 71 Stanford Law Review Online. 2018

[25] United Nations. Declaration on the elimination of violence against women. 1993. https://www.un.org/en/genocideprevention/documents/atrocity-crimes/Doc.21_declaration%20elimination%20vaw.pdf

[26] Coalition of Feminists for Social Change (COFEM). Connecting gender-based violence, sexual harassment and everyday sexism. Feminist Pocket Book. TIP SHEET #3. 2018. https://cofemsocialchange.org/wp-content/uploads/2018/11/TS3-Connecting-gender-based-violence-sexual-harassment-and-everyday-sexism.pdf

[27] Berdahl JL (2007). Harassment based on sex: Protecting social status in the context of gender hierarchy. The Academy of Management Review.

[28] Advocates for Human Rights. Sexual harassment and the subordination of women. http://www.stopvaw.org/Sexual_Harassment_and_the_Subordination_of_Women. Accessed Feb 2020.

[29] Stockdale MS, Berry CG, Schneider RW, Cai F (2004). Perceptions of the sexual harassment of men. Psychol Men Masculinity.

[30] Berdahl J, Magley VR, Waldo C (1996). The sexual harassment of men?: Exploring the concept with theory and data. Psychol Women Quar.

[31] Knapp DE, Kustis GA (2000). Same-sex sexual harassment: A legal review with implications for organizational policy. Employee Responsibilities and Rights Journal.

[32] Fitzgerald LF, Gelfand MJ, Drasgow F (1995). Measuring sexual harassment: Theoretical and psychometric advances. Basic Appl Soc Psychol.

[33] Schultz V (1998). Reconceptualizing sexual harassment. The Yale Law Journal.

[34] Schultz V. Reconceptualizing sexual harassment, again. SSRN Journal. 2018.

[35] National Academies of Sciences, Engineering, and Medicine; Policy and Global Affairs; Committee on Women in Science, Engineering, and Medicine; Committee on the Impacts of Sexual Harassment in Academia; Benya FF, Widnall SE, Johnson PA, editors. Washington (DC): National Academies Press (US); 2018 29894119

[36] Leskinen EA, Cortina LM, Kabat DB. Gender harassment: Broadening our understanding of sex-based harassment at work. *Law and Human Behavior.* 2010. 10.1007/s10979-010-9241-5 20661766

[37] Newman C, Ng C, Pacqué-Margolis S, Frymus D (2016). Integration of gender-transformative interventions in health professional education reform for the 21^st^ century. Human Resourc Health.

[38] Newman C, De Vries D, Kanakuze J, and Ngendahimana G. Workplace Violence and Gender Discrimination in Rwanda’s Health Workforce: Increasing Safety and Gender Equality. Human Resources for Health. 2011. Vol. 9. 10.1186/1478-4491-9-19 PMC3154143 21767411

[39] Newman C, Chama PK, Mugisha M, Matsiko CW, Oketcho V. Reasons behind the current gender imbalance in senior global health roles and the practice and policy changes that can catalyze organizational change in gendered organizations. Global Health, Epidemiology and Genomics. Global Health, Epidemiology and Genomics. 2017. Volume 2, e19. 10.1017/gheg.2017.11 PMC5870424 29868225

[40] Newman C (2014). Time to address gender discrimination and inequality in the health workforce. Human Resourc Health.

[41] Newman C, Kimeu A, Penders C, Shamblin L, Bwonya J, McQuide P (2011). Making nondiscrimination and equal opportunity for education a reality in Kenya’s pre-service health training institutions: results of a gender analysis. World Health and Population..

[42] International Labour Organization. Violence and Harassment Convention 190. Article 1. 2019.https://www.ilo.org/dyn/normlex/en/f?p=NORMLEXPUB:12100:0::NO::P12100_ILO_CODE:C190

[43] Olney S. ILO Convention on Violence and Harassment: Five key questions. International Labour Organization. https://www.ilo.org/global/about-theilo/newsroom/news/WCMS_711891/lang--en/index.htm. Accessed May 2020

[44] Berdahl JL (2007). The Sexual harassment of uppity women. J Appl Psychol.

[45] International Labour Organization, International Council of Nurses, World Health Organization and Public Services International. Framework guidelines for addressing workplace violence in the health sector. Geneva, International Labour Office, 2002. https://apps.who.int/iris/handle/10665/42617

[46] di Martino V. Workplace violence in the health sector: Country case studies Brazil, Bulgaria, Lebanon, Portugal, South Africa, Thailand and an additional Australian study: Synthesis report. Geneva, Switzerland: ILO/ICN/WHO/PSI Joint Programme on Workplace Violence in the Health Sector, 2002. http://www.who.int/violence_injury_prevention/violence/activities/workplace/WVsynthesisreport.pdf

[47] Chaudhuri, P. Sexual harassment in the workplace: Experiences of women in the health sector. Health and Population Innovation Fellowship Programme Working Paper No. 1. New Delhi: Population Council 2006.

[48] Cantalupo, NC, Kidder, WC. Sexual harassment of students by university faculty: A systematic look at a serial problem. Utah Law Review. Volume 2018. Number 3. Article 4.

[49] Sen P, Borges E, Guallar E, Cohran J. Towards an end to sexual harassment: The urgency and nature of change in the era of #MeToo. Office of the Executive Coordinator and Spokesperson on Addressing Sexual Harassment and Discrimination at UN Women. 2018.

[50] European Union Agency for fundamental rights. Violence against women: an EU survey. Main results. 2014.p. 95-96. https://fra.europa.eu/en/publication/2014/violence-against-women-eu-wide-survey-main-results-report

[51] Hlavka HR (2014). Normalizing sexual violence: Young women account for harassment and abuse. Gender Soc.

[52] Mateo A, Menza K. The results of a 1976 survey of women about sexual harassment at work remain virtually unchanged in 2017. Redbook. 2017. https://www.Redbookmag.Com/Life/Money-Career/A49220/Sexual-Harassment-In-The-Workplace/. Accessed Febr 2020.

[53] Dobbin F, Kalev A. Why diversity programs fail. Harvard Business Review. 2016.

[54] McLaughlin H, Uggen C, Blackstone A (2017). The economic and career effects of sexual harassment on working women. Gend Soc..

[55] Schneider KT, Swan S, Fitzgerald LF (1997). Job-related and psychological effects of sexual harassment in the workplace: empirical evidence from two organizations. J Appl Psychol..

[56] Willness CR, Steel P, Lee K. A Meta-Analysis of the Antecedents and Consequences of Workplace Sexual Harassment. Personnel Psychology. 2007;60(1):127–62. /10.1111/j.1744-6570.2007.00067.x

[57] Feldblum CR, Lipnic VA. Select Task Force on the Study of Harassment in the Workplace: Report of Co-Chairs. 2016. https://www.eeoc.gov/eeoc/task_force/harassment/report.cfm

[58] Suarez E, Gadalla TM (2010). Stop blaming the victim: a meta-analysis on rape myths. J Interpers Viol.

[59] Chapleau KM, Oswald DL, Russell, BL. Male rape myths: The role of gender, violence and sexism. Journal of Interpersonal Violence. 2008. 10.1177/0886260507313529 18259049

[60] Fitzgerald L (2017). Still the last great open secret: Sexual harassment as systemic trauma. J Trauma Dissoc.

[61] Baugh SG (1997). On the persistence of sexual harassment in the workplace. J Bus Ethics.

[62] Katz J (2006). The macho paradox: Why some men hurt women and how all men can help.

[63] Freyd JJ. What is DARVO? https://dynamic.uoregon.edu/jjf/defineDARVO.html. Accessed Sept 2019.

[64] Abramson K (2014). Turning up the lights on gaslighting. Philos Persp..

[65] Gaslighting SC (2019). Misogyny and psychological oppression. Monist.

[66] Ahern K (2018). Institutional betrayal and gaslighting: Why whistle-blowers are so traumatized. Continuing education. Perinat Neonat Nurs..

[67] Wear D & Altman J. Sexual harassment in academic medicine: Persistence, non-reporting, and institutional response. Med Educ Online. 2005:10:10. http://www.med-ed-online.org. 10.3402/meo.v10i.4377 28253151

[68] Hinze SW (2004). “Am I being oversensitive?” Women’s experience of sexual harassment during medical training. health: an Interdisciplinary. J Soc Study Health Illn Med..

[69] Gioia DA, Corley KG, Hamilton AL (2013). Seeking qualitative rigor in inductive research: Notes on the Gioia methodology. Org Res Meth.

[70] Uganda Ministry of Health. Guidelines to implement the policy on prevention and response to sexual harassment. 2018. http://library.health.go.ug/publications/gender-based-violence/guidelines-implement-policy-prevention-and-response-sexual

[71] Manne K (2017). Down Girl: The logic of misogyny.

[72] Boland, ML. Sexual harassment: Your guide to legal action. What you should know and what you can do. Sphinx Publishing: Napierville: 2002 (Quoting the decision on Meritor v. Vinson]

[73] International Association of Women Judges. Stopping the abuse of power through sexual exploitation: Naming, shaming, and ending sextortion. 2012. Washington, D.C.: Frank Vohl. http://www.iawj.org/wp-content/uploads/2017/04/Corruption-and-Sextortion-Resource-1.pdf

[74] Kabat-Farr D, Crumley ET (2019). Sexual harassment in healthcare: a psychological perspective. Online J Issues Nurs..

[75] Advocates for Human Rights. Violence and power. http://stopvaw.org/Violence_and_Power. 2018. Accessed Feb 2020.

[76] Bergman ME, Langhout RD, Palmieri PA, Cortina LM, Fitzgerald LM. The (un)reasonableness of reporting: Antecedents and consequences of reporting sexual harassment. J Appl Psychol Copyright 2002 by the American Psychological Association, Inc. 2002, Vol. 87, No. 2, 230–242. 10.1037/0021-9010.87.2.230 12002952

[77] Precious T. New workplace sexual harassment protections become law. buffalonews.com. Accessed Dec 2020

[78] Ayres I & Unkovic C. Information Escrows. 111 Mich. L. Rev.145 (2012). https://repository.law.umich.edu/mlr/vol111/iss2/1.

[79] Dobbin F, Kalev A. Training programs and reporting systems won’t end sexual harassment. Promoting women will. Harvard Business Review. 2017.

[80] Schulte, Brigid. To combat harassment, more companies should try bystander training. Harvard Business Review. 2018.

[81] Swann, SL. Bystander interventions. Wisconsin Law Review. 2015:975–1047

[82] Kahsay WG, Negarandeh R, Dehghan N, Hasanpour M (2020). Sexual harassment against female nurses: a systematic review. BMC Nurs.

[83] Rimmer A. BMA sexism: Doctors at annual meeting hear fresh allegations of harassment. BMJ. 2019. 10.1136/bmj.l4455 31248944

[84] United Nations Human Rights Office of the High Commissioner. CEDAW General Recommendation 35 on gender-based violence against women. July 2017. Treaty bodies Download (ohchr.org). Accessed December 2020.

[85] United Nations General Assembly: Report of the Working Group on the Issue of Discrimination Against Women in Law and Practice. Human Rights Council; 2012. A/HRC/20/28. [http://www.ohchr.org/Documents/Issues/Women/AHRC-20-28_en.pdf].

[86] Kasperkevic, J. HR is not there to be your friend. It’s there to protect the company. Marketplace. 2017. Accessed Dec 2020.

[87] United Nations Human Rights Office of the High Commissioner. Optional Protocol to the Convention on the Elimination of All Forms of Discrimination against Women. OHCHR | Optional Protocol CEDAW. Accessed December 2020.

[88] International Labor Organization. Decent work. Decent work (ilo.org) accessed January 2021.

[89] International Peace Institute.Combatting sexual harassment at the United Nations. March 29, 2018. Combating Sexual Harassment at the United Nations | International Peace Institute (ipinst.org). Accessed Feb 2021.

[90] Hossain N, Musembi CN and Hughes, J. Corruption, accountability and gender: Understanding the connections. Primers in gender and democratic governance. UNDP and UNIFEM. 2010. Corruption-accountability-and-gender(9).pdf. Accessed Feb 2021.

[91] United Nations Development Program. Fighting corruption in the education sector: Methods, tools and good practices. UNDP. 2011. Anticorruption Methods and Tools in Education Lo Res (1).pdf. Accessed Feb 2021.

