# Supplementary material for: Uganda’s response to sexual harassment in the public health sector: from “Dying Silently” to gender-transformational HRH policy
Source: Hum Resour Health. 2021 May 1;19:59. doi: 10.1186/s12960-021-00569-0 (PMC8087889; doi:10.1186/s12960-021-00569-0)
Supplement: Supplementary file 1 — Additional file 1: Illustrative Definitions. [file 12960_2021_569_MOESM1_ESM.docx]

| **Additional File 1: Illustrative Definitions** [References at the end] | |
| --- | --- |
| **Gender-based violence** [1] | Violence that is directed against a woman because she is a woman or that affects women disproportionately. It includes acts that inflict physical, mental or sexual harm or suffering, threats of such acts, coercion and other deprivations of liberty. A form of discrimination that seriously inhibits women’s ability to enjoy rights and freedoms on a basis of equality with men.  17. Equality in employment can be seriously impaired when women are subjected to gender-specific violence, such as sexual harassment in the workplace.  18. Sexual harassment includes such unwelcome sexually determined behaviour as physical contact and advances, sexually-coloured remarks, showing pornography and sexual demands, whether by words or actions. Such conduct can be humiliating and may constitute a health and safety problem; it is discriminatory when the woman has reasonable ground to believe that her objection would disadvantage her in connection with her employment, including recruitment or promotion, or when it creates a hostile working environment. |
| **Psychological violence** [2] | Intentional use of power, including threat of physical force, against another person or group, that can result in harm to physical, mental, spiritual, moral or social development. It includes verbal abuse, bullying/ mobbing, harassment and threats. |
| **Violence and harassment** [3] | (a) The term “violence and harassment” in the world of work refers to a range of unacceptable behaviors and practices, or threats thereof, whether a single occurrence or repeated, that aim at, result in, or are likely to result in physical, psychological, sexual or economic harm, and includes gender-based violence and harassment.  (b) The term “gender-based violence and harassment” means violence and harassment directed at persons because of their sex or gender, or affecting persons of a particular sex or gender disproportionately, and includes sexual harassment. |
| **Workplace violence** [4] | Incidents where staff are abused, threatened or assaulted in circumstances related to their work, including commuting to and from work, involving an explicit or implicit challenge to their safety, well-being or health. Examples include physical attack, verbal abuse, sexual harassment, racial harassment and bullying. |
| **Sexual harassment** [5] | Any unwanted, unreciprocated and unwelcome behaviour of a sexual nature that is offensive to the person involved, and causes that person to feel threatened, humiliated or embarrassed. Can include both physical and psychological violence, in combination with other forms of workplace violence (e.g., verbal abuse, bullying, racial harassment). Racial harassment may overlap and include threatening conduct that is based on race, ethnicity, color, language, national origin, religion, association with a minority, birth or other status that is unreciprocated or unwanted and which affects the dignity of women and men at work. |
| **Sexual harassment** [6] | A form of discrimination composed of three categories of behavior (1) gender harassment (verbal and nonverbal behaviors that convey hostility, objectification, exclusion, or second-class status about members of one gender); (2) unwanted sexual attention (verbal or physical unwelcome sexual advances, which can include assault); and (3) sexual coercion (when favorable professional or educational treatment is conditioned on sexual activity). Harassing behavior can be either direct (targeted at an individual) or ambient (a general level of sexual harassment in an environment). It constitutes discrimination because it is harmful and it is based on gender—it is not necessarily motivated by sexual desire nor does it need to involve sexual activity. |
| **Sexual harassment** [7] | A human rights violation of gender-based discrimination, regardless of sex, in a context of unequal power relations such as a workplace and/or gender hierarchy. Sexual harassment in employment as unwelcome sexual conduct in the world of work. The experience comes in two types: (1) an environmental workplace condition, made up of persistent or pervasive conduct or a single serious incident, or (2) proposal or execution of an exchange of a workplace benefit or absence of workplace detriment for sexual compliance. Each type is equally grave. Sexual conduct, to be harassment, is unwelcome. Unwelcomeness is the standard used, not “consent”. |
| **Sexual harassment** [8] | A manifestation of sex-based discrimination, the ILO defined sexual harassment as a sex-based behaviour that is unwelcome and offensive to its recipient. It may take two forms:1) Quid Pro Quo, when a job benefit - such as a pay rise, a promotion, or even continued employment - is made conditional on the victim acceding to demands to engage in some form of sexual behaviour; or; 2) hostile working environment in which the conduct creates conditions that are intimidating or humiliating for the victim: |
| **Sexual harassment** [9] | Unwelcome sexual advances, requests for sexual favors, and other verbal or physical conduct of a sexual nature constitutes sexual harassment when submission to or rejection of this conduct explicitly or implicitly affects an individual's employment, unreasonably interferes with an individual's work performance or creates an intimidating, hostile or offensive work environment. |
| **Sex-based harassment (**Drawn from [6] and [10]) | Encapsulates a wide range of behaviors that degrade or humiliate an individual based on their sex and/or gender. Three different categories of behavior define sex-based harassment: (1**) “gender harassment**,” referring to sexist verbal and nonverbal behaviors that convey hostility, objectification, exclusion, or second-class status about members of one gender. For example, behaviors that demean women and/or femininity or create a hostile work environment, but which do not have the goal of sexual cooperation. Can also function to police gender ideals. (2) “**unwanted sexual attention,”** or verbal or physical unwelcome sexual advances which can include assault, referring to behaviors such as pressure for dates and unwanted touching which express a romantic or sexual interest but are unreciprocated and unwelcome; and  (3) **“sexual coercion,”** when favorable professional or educational treatment is conditioned on sexual activity, includes behaviors that threaten loss of job, unfavorable work assignments, or loss of pay or promised promotion, raises, or better assignments in return for sexual cooperation. Harassing behavior can be either direct (targeted at an individual) or ambient (a general level of sexual harassment in an environment). It constitutes discrimination because it is harmful and it is based on gender —it is not necessarily motivated by sexual desire nor does it need to involve sexual activity. |
| **Sexual abuse and exploitation** [11] | **Abuse** is the actual or threatened physical intrusion of a sexual nature whether by force or under unequal or coercive conditions. **Exploitation** is the actual or attempted abuse of a position of vulnerability, differential power or trust for sexual purposes, including but not limited to profiting monetarily, socially or politically from the sexual exploitation of another. |
| **Sextortion** [12] | Abuse of a position of authority or power to extort sex. Conduct that is not only sexually abusive and exploitative but contains an element of corruption. Three elements (1) Abuse of legitimate authority; (2) *Quid pro quo* (this for that) exchange wherein a benefit or detriment is conditioned on the employees response to a sexual request ; and (3) Psychological coercion to obtain sexual favors. Functions similarly to the widely known form of corruption, fraud and financial bribes; however, instead of money or goods, sexual demands are the “currency” for this kind of corruption. It involves a breach of trust by actors who have abused the social power derived from their position of doctor, teacher, supervisor or cleric. When a person in a position of authority demands a sexual favor in return, it is sextortion. |

1. United Nations. General Recommendation 19 to CEDAW on violence against women. <https://www.refworld.org/docid/52d920c54.html>

2. ILO/ICN/WHO/PSI. Framework guidelines for addressing workplace violence in the health sector. Geneva, Switzerland. ILO/ICN/WHO/PSI Joint Programme on Workplace Violence in the Health Sector, Geneva, International Labour Office. 2002. [http://www.ilo.org/public/english/dialogue/sector/papers/health/guidelines.pdf](http://www.jil.go.jp/bulletin/year/1997/vol36-09/06.htm)

3. International Labour Organization. Violence and Harassment Convention 190. Article 1. 2019. <https://www.ilo.org/dyn/normlex/en/f?p=NORMLEXPUB:12100:0::NO::P12100_ILO_CODE:C190>

4. ILO/ICN/WHO/PSI. 2002

5. ILO/ICN/WHO/PSI. 2002

6. National Academies of Sciences, Engineering, and Medicine. Sexual harassment of women: Climate, culture, and consequences in academic sciences, engineering, and medicine. 2018. Washington, DC: The National Academies Press. p.25. <https://doi.org/10.17226/24994>.

7. Office of the Executive Coordinator and Spokesperson on Addressing Sexual Harassment and Discrimination at UN Women. Sen P, Borges E, Guallar E, Cochran J. Towards an end to sexual harassment: The urgency and nature of change in the era of #MeToo. UN Women, 2018.

8. International Labor Office. Sexual Harassment at Work Fact Sheet. Work in Freedom. <https://www.ilo.org/wcmsp5/groups/public/---ed_norm/---declaration/documents/publication/wcms_decl_fs_96_en.pdf>.

9. US Equal Opportunity Employment Commission. Facts about Sexual Harassment. <https://www.eeoc.gov/fact-sheet/facts-about-sexual-harassment>

10. Leskinen EA, Cortina LM, Kabat DB. Gender harassment: Broadening our understanding of sex-based harassment at work. *Law and Human Behavior.* 2010, <https://lsa.umich.edu/psych/lilia-cortina-lab/Leskinen%20et%20al.%202010%20LHB.pdf>

11. United Nations. Secretary General’s Bulletin. Special measures of protection from sexual exploitation and abuse .SGB/2003/13. <https://reliefweb.int/report/world/secretary-generals-bulletin-special-measures-protection-sexual-exploitation-and-sexual>

12. International Association of Women Judges. Stopping the abuse of power through sexual exploitation: Naming, shaming, and ending sextortion. 2012. Washington, D.C.: Frank Vohl. <http://www.iawj.org/wp-content/uploads/2017/04/Corruption-and-Sextortion-Resource-1.pdf>. Also see: Tanzanian Women Judges Association. Stopping the abuse of power for purposes of sexual exploitation: Naming, shaming and ending sextortion: A toolkit.
