# Supplementary material for: Uganda’s response to sexual harassment in the public health sector: from “Dying Silently” to gender-transformational HRH policy
Source: Hum Resour Health. 2021 May 1;19:59. doi: 10.1186/s12960-021-00569-0 (PMC8087889; doi:10.1186/s12960-021-00569-0)
Supplement: Supplementary file 2 — Additional file 2: Definition and Interpretation from Uganda’s 2012 Employment (Sexual Harassment) Regulations. [file 12960_2021_569_MOESM2_ESM.docx]

| **Additional File 2: Definition and Interpretation from Sections of Uganda’s 2012 Employment (Sexual Harassment) Regulations** |
| --- |
| *“[1.2]. “Harassment” means verbal or physical abuse or behaviour that unreasonably interferes with work or creates an intimidating, hostile, or offensive work environment including intimidation;*  *“Intimidation” means physical or verbal abuse, or behaviour directed at isolating or humiliating an individual or a group or at preventing them from engaging in normal activities and includes—*   1. *degrading public tirades by a supervisor or colleague;* 2. *deliberate insults related to a person's personal or professional competence;* 3. *threatening or insulting comments, whether oral or written including by e-mail; and* 4. *deliberate desecration of religious or national symbols or both.*   *“Retaliation” means any unwarranted action against an employee or employer or any other person who may be involved in a sexual harassment complaint;*  *“Sexual harassment in employment” means—*   1. *a direct or implicit request to an employee for sexual intercourse, sexual contact or any other form of sexual activity that contains—* 2. *an implied or express promise of preferential treatment in employment;* 3. *an implied or express threat of detrimental treatment in employment;* 4. *an implied or express threat about the present or future employment status of the employee;* 5. *use of language whether written or spoken of a sexual nature such as unwelcome verbal advances, sexual oriented comments, request for sexual favours, jokes of a sexual nature, offensive flirtation or obscene expressions of sexual interest that are addressed directly to the person;* 6. *use of visual material of a sexual nature such as display of sexually suggestive pictures, objects or written materials or sexually suggestive gestures; and* 7. *showing physical behavior of a sexual nature such as unwanted and unwelcome touching, patting, pinching or any other unsolicited physical contact;*   *which directly or indirectly subjects the employee to behaviour that is unwelcome or offensive to that employee and that, either by its nature or though repletion, has a detrimental effect on that employee’s employment, job performance or job satisfaction.*  *[5]. Prohibition of the dissemination of sexual materials.*  *An employer shall specifically prohibit the dissemination of sexual explicit voice mail, e-mail, graphics, downloaded material or websites in the workplace and shall include these prohibitions in the workplace policy.*  *[17] Retaliation and discrimination.*   1. *A person involved in a sexual harassment complaint under these Regulations shall not be retaliated against for doing the following—* 2. *consulting on, reporting or filing a complaint of sexual harassment;* 3. *testifying as a witness in a claim of sexual harassment;* 4. *cooperating during any investigation of a sexual harassment complaint;* 5. *participating in a meeting constituted to discuss sexual harassment in the workplace;* 6. *discussing the complaint of sexual harassment with the labour union representatives or the employers’ organizations; and* 7. *carrying out any duties as a member of the committee on sexual harassment.*   *[7.17.1] An employer shall not discriminate against an employee based on the employee’s involvement in a sexual harassment complaint. Discrimination under this regulation includes—*   1. *termination;* 2. *denial of promotion;* 3. *demotion in title or duties;* 4. *transfer to a less favorable position or location;* 5. *involuntary placement on leave;* 6. *hostile or abusive treatment;* 7. *decreasing remuneration or benefits;* 8. *coercion;* 9. *threats; and* 10. *intimidation.* |
| **Employment (Sexual Harassment) Regulations, 2012. The Uganda Gazette No. 21 Volume CIV dated 20th April, 2012 Entebbe.** [**https://ulii.org/ug/legislation/statutory-instrument/2012/15**](https://ulii.org/ug/legislation/statutory-instrument/2012/15) |
