# Supplementary material for: Uganda’s response to sexual harassment in the public health sector: from “Dying Silently” to gender-transformational HRH policy
Source: Hum Resour Health. 2021 May 1;19:59. doi: 10.1186/s12960-021-00569-0 (PMC8087889; doi:10.1186/s12960-021-00569-0)
Supplement: Supplementary file 3 — Additional file 3: Data Collection Methods and Sample. [file 12960_2021_569_MOESM3_ESM.docx]

**Additional File 3: Data Collection Methods and Sample**

The objectives of the Uganda MOH formative assessment were: Describe the forms of, contributors and reactions to and consequences of sexual harassment in government health workplaces; identify the gender dynamics of sexual harassment; identify implementation challenges and opportunities relative to sexual harassment law and policy; assess the adequacy and implementation of existing policy and law related to sexual harassment; and recommend elements of an effective sexual harassment prevention and response system. Mixed methods were used to collect the data and results triangulated to aid the interpretation of results. The methods included document review, Key informant and in-depth interviews, focus group discussions and observations.

**1) Document Review:** Document review of existing legal and policy guidance related to health worker conduct or to sexual harassment, to facilitate a broader understanding of the organizational and societal context and aspirations for the regulation of sexual harassment, and of the adequacy of existing policy and law related to sexual harassment. The documents were reviewed using a standardized *“Policy-Legal Document Review Form”*. See Table 1, below for the documents reviewed.

2) **Focus group discussions:** Focus group discussions were conducted in two phases with health workers and managers at hospitals and health center IIIs and IVs. Data were collected in two phases/stages for the following reasons:

1. Phase 1 FGD data are needed to understand sexual harassment in Ugandan public sector health workplaces.
2. Phase 2 FGD participants and in-depth interview informants (using an In-Depth Interview Guide) were recruited from the Phase 1 FGD participants.

***Phase 1: Focus Group Discussions***

1. ***Single sex focus group discussions* (See Appendix B1).** FGDs were conducted with male and female health workers and managers in same-sex groups at Health Centre (HC) III and HC IV and hospital facilities to elicit the forms of, reactions to and impacts of sexual harassment, including reporting constraints. Gender-sensitive research suggests that communication around gender norms and perceptions of gender issues may be facilitated in same-sex groups because of an assumption of shared or similar experience. Each group of six persons was homogeneous and representative of health workers in line (i.e., non-management) and management positions. FGD members shared similar characteristics such as line or management organizational status, urban/rural residence, language level and education. The participants from the different health centre IIIs would meet at a health centre IV to participate in the FGDs. In total, 15 FGDs were conducted: three (two female and one male) in five districts: Gulu, Hoima, Mukono, Rukungiri and Tororo.

**3) Key Informant Interviews:** Key informant interviews were conducted with people knowledgeable about sexual harassment in Uganda and with the operationalization of national policy and law and existing systems. In total 22 key informant interviews were conducted at national and district levels. At the national level, key informants came from the Ministry of Health, the Ministry of Gender, Labor and Social Development, the Ministry of Public Service, the Equal Opportunity Commission, health professional councils, Makerere University, women’s groups at national levels, and individuals known for their experience in the field of sexual harassment in Uganda. At the district level, key informants were drawn from District Health Management Teams and hospital administration.

**4) Baseline Survey:** Baseline information on specific measures of a sexual harassment prevention and response system were collected from facility-level health workers and managers—the ultimate users and beneficiaries of the system. A set of closed-ended questions were administered to 60 health workers and managers working at the service facilities in all ten priority districts. The survey collected baseline information on measures of a sexual harassment prevention and response system at the facility level, including:

- Knowledge of sexual harassment;
- Availability of policy or code of conduct against sexual harassment;
- Managers and health workers trained to recognize, report and resolve sexual harassment;
- Accessible information on reporting and investigating procedures for sexual harassment;
- Availability of communications materials, such a wall poster on zero tolerance for sexual harassment, or a “Know Your Rights” type of booklet for health workers;
- A confidential system of reporting/investigation/resolution described and in place at decentralized levels; and,
- Statistics on or reports of sexual harassment claims investigated, documented and successfully resolved.

**Phase 2 Data Collection**

**1) Mixed Sex, Follow-Up Focus Group Discussion:** FGD data collection in this phase examined the extent to which sexual harassment is recognized, named, problematized differently depending on gender and organizational status. Gender differences in beliefs and perceptions may contribute to interaction dynamics that obstruct clear and collaborative communication about sexual harassment and about recommended systems or strategies to prevent and regulate it. The mixed-sex focus groups with health workers and managers shed light on the gendered communication around sexual harassment.

One mixed-sex FGD was conducted in five districts: Gulu, Hoima, Mukono, Rukungiri and Tororo**.** Each FGD had six to eleven participants. Phase 2 FGD participants and in-depth interview informants were recruited from the Phase 1 FGD participants. At the end of each of the Phase 1 same-sex FGDs, the data collectors invited some of the Phase 1 FGD participants to take part in the Phase 2 mixed-sex follow-up FGDs or in-depth interviews during which they discussed gender dynamics involved in sexual harassment in more detail. Selection criteria were willingness to actively participate in a follow-up FGD/interview, ability to be articulate and to “hold their own” in a mixed group, and whether they had a story to tell. Six focus group discussions were also conducted with managers in five districts: Gulu, Hoima, Rukungiri, Mukono and Tororo.

**2) In-Depth Interviews (IDIs):** IDIs were conducted with volunteers recruited from phase 1 FGDs and key informants to elicit detailed descriptions of experiences of sexual harassment, since informants might feel more willing to disclose in privacy. Eight IDIs were conducted with six female volunteers and two with male volunteers from different line and management levels of the health workforce.

**3) Follow-Up Exercise:** Once the preliminary results were in, follow-up visits were made to ten sites to interview In-Charge Nurses where sexual harassment of patients had emerged from FGDs. Also, a checklist with indicators of “indecent dressing” was tested.

A summary of the data collection methods and sources are presented in Table 1.

| **Additional File 3, Table 1: Uganda Ministry of Health Sexual Harassment Formative Assessment: Data Collection Methods and Sources** | | |
| --- | --- | --- |
| **Phase 1 Method** | **Purpose/Objective(s)** | **Sources** |
| **Baseline Survey** | Questionnaire with closed- and open-ended items establish baseline on essential measures of success of a sexual harassment prevention and response system for the health sector: definition of sexual harassment; available policy or code of conduct; training; communications materials; recording system. Administered in a one-to-one interview. | **60 health workers**   - 35 (58 %) frontline health workers - 25 (42 %) facility managers |
| **Document Review** | Three- part form with criteria assessing the policy/law’s content, implementation guidance and enforcement measures. Elicits the larger organizational and societal context and aspirations for the prevention and regulation of sexual harassment. | **5 documents**   - Ministry of Gender, Labor and Social Development’s 2012 *Sexual Harassment Regulations* - Ministry of Health’s 2014 *Guidelines for Mainstreaming Gender in Human Resources for Health Management* - Ministry of Public Service’s 2005 *Code of Conduct and Ethics for Uganda Public Service* - Ministry of Health’s 2008 *Guidelines for Occupational Safety and Health (OSH), Including HIV in the Health Services Sector* - Ministry of Health’s *2009 Joint Code of Conduct and Ethics for Health Workers in Uganda* |
| **Single Sex Focus Group Discussion** | Elicit frontline and management-level health workers’ perceptions and experience of sexual harassment in health workplaces. Describes the **forms, contributors**, **reactions** to and **impacts** of sexual harassment, including contextual factors**, reporting patterns** and **constraints;** and the **recommended elements** of an effective sexual harassment prevention and response system for the health sector. | **15 groups with 5-11 participants in each, total participants 106**   - 73 (69%) female - 33 (31%) male |
| **Managers Mixed Sex FGD** | Elicit management-level perceptions and experience of sexual harassment in health workplaces. Describes the **forms, contributors**, **reactions** to and **impacts** of sexual harassment, including contextual factors**, reporting patterns** and **constraints;** and the **recommended elements** of an effective sexual harassment prevention and response system for the health sector | **6 groups mixed sex, total participants 39**  20 (51.3%) female  19 (48.7%) male |
| **Key Informant Interview** | Assess HRM and sexual harassment stakeholders’ understanding of sexual harassment in Uganda health workplaces and the status of legal and policy measures to prevent and regulate sexual harassment, including: the current implementation status of available legal-policy guidelines relevant to sexual harassment at national, regional and district levels in the health sector, including policy feasibility; the implementation gaps, challenges and opportunities related to sexual harassment law and policy; and recommended elements of an effective sexual harassment prevention and response system for the health sector. | **25 key informants**  National level (15 national, including MOH, MOGLSD, MOPS, Makerere University, Uganda Nurses Union, UWONET, UMDPC, EOC, National Organization for Trade Unions, COFTU, Association of Women’s Judges, other knowledgeable individual informants; and district-level informants (e.g., District Health Officers [DHOs]).   - 10 (40%) female - 15 (60%) male |
| **Phase 2 Method** | **Purpose/Objective(s)** | **Sources** |
| **Mixed Sex Focus Group Discussion with case scenarios and observation form** | Elicit health workers’ perceptions and experience of sexual harassment in health workplaces, especially whether sexual harassment is **recognized**, **named, problematized** differently depending on gender; and key **gendered power dynamics** involved in sexual harassment, including patterns of **interaction** and **communication** between men and women around the issue of sexual harassment. Also, the recommended **elements** of an effective sexual harassment prevention and response **system** for the health sector. | **5 groups with 6-11 participants in each, total participants 38**   - 26 (68%) female - 12 (32%) male |
| **In-Depth Interviews** | Elicit detailed information based on personal knowledge of sexual harassment in public health sector workplaces, either experienced or witnessed, including the type of harassment, its perceived harms/consequences, gendered power dynamics involved in the experience of sexual harassment, victim or witness reactions, disclosing or reporting the harassment; and recommended elements of an effective sexual harassment prevention and response system for the health sector. | **10 health workers**   - 8 (80%) female - 2 (20%) male |
| **Follow-Up Exercise:**  **Interviews** | Elicit information to verify data from focus group discussions on the sexual harassment of patients by health workers | **16 health workers in 8 health facilities**  4 (25%) Male  12 (75%) Female |
