# Supplementary material for: Uganda’s response to sexual harassment in the public health sector: from “Dying Silently” to gender-transformational HRH policy
Source: Hum Resour Health. 2021 May 1;19:59. doi: 10.1186/s12960-021-00569-0 (PMC8087889; doi:10.1186/s12960-021-00569-0)
Supplement: Supplementary file 4 — Additional file 4: Forms and Examples of Sexual Harassment Experienced by Health Employees. [file 12960_2021_569_MOESM4_ESM.docx]

| **Additional Data File 4: Forms and Examples of Sexual Harassment Experienced by Health Employees** (From FGDs and KIIs) | |
| --- | --- |
| **Quid pro quo)** | **Hostile environment** |
| - **Aggressive pressure, bullying, intimidation**   *“A* ***boss*** *who wants to work with you all the time assigning you duties which are not yours so that he may have sex with you, and he tells you to go with him to the ward round when you are not supposed to be there, or to do a certain procedure.”*  *“Some start hitting on you through* ***abuses and criticism*** *that you are lazy at work yet it is not true, but they do that with a hidden agenda. With time people realize that they no longer* ***abuse*** *you and wonder why it is the case. When you give in the* ***abuses*** *stop.”*  Using superior power, strength or influence to intimidate, coerce, frighten or overawe (someone), typically to force her/him to do what one wants):  *“Use of* ***threats*** *of transfer by top supervisors for example if you don’t give me something then I will deal with you”*   - **Bribery** (Offering something valuable (such as money) that is given in order to get someone to do something)   *“There is also someone* ***giving you money*** *and by the time someone realizes what the money is for, he is already demanding for sex. “*  *“Because you know if the person told you to do this, you will give in because they know you want some money.”*   - **Seduction** (Use something to attract, entice or tempt to sexual relations)   *“A boss or a colleague can start giving you motivation in form of money as a* ***token*** *of appreciation and in the end he asks for sex”*  *“We have our seniors… when you go to them to show you how to write a report, you find someone telling you that if you want me to show you, come over to my place and we discuss it, or let us go to the hotel and we discuss that issue.”*   - **Explicit/implicit promise of career advancement/training in exchange of sexual favors**   “*I had forgotten this case. There was a nurse here and she wanted to go and do other training. I was her supervisor and I recommended her to go but then I was not the final person to authorize. There were two nurses and so my boss authorized one and left the other. But because I was the direct supervisor, I did not know why and so she came crying to me saying that ‘your friend is doing this to me, that before I go to school, I must do this.’ Serious, she went to school but when she came back, she did not stay. She told me that ‘I* ***cannot continue*** *working here’ (and) she now works in Buwendo hospital.”*  *“To add on what my sister has said in the field of employment, you find that in some offices, to go for training, they will ask for sex.”*  *“There was a nurse who wanted to go for further studies, since I was the final person, I had authorized her to go but her immediate supervisor was not willing to let go. I came to realize he was making sexual advances and because of this, when she finished her studies, she* ***changed*** *her station.”*   - **Explicit/implicit promise of recruitment, promotion or pay raise in exchange of sexual favors; or threat to withhold**   *“Promotion promises or actual promotions which they will use later to actualize their actions. You may be made the head of OPD but his intentions are to call you at any time so that he can be with you. “*  *“Sexual harassments usually happens during times when they have promoted people, somebody can first ask, can I sleep with you before we give you a* ***promotion****?”*  *“For others, they are told in order to be promoted, you have to first have to do this and that. If you don’t give in, you are* ***not promoted***.”  *“Some people work as volunteers and some health workers tell them that they are friends with the administration and that they can fix them in the hospital. They tell her that before doing that for you, you have to first do this for me so that I help you and fix you in so that you start* ***getting a salary*** *also.* “  *“The first thing is, for you to get employment, some officer may ask for sex. For you to be promoted, they will ask for sex.”*  *“Another form is about we the health workers, we have got a lot of our desires which are so high especially when it comes to issues of* ***promotion.*** *If I want to be promoted from the nursing officer to another rank (position), assuming it’s the nurse requesting promotion from a male doctor, then there shall be a serious discussion between them the doctor may ask, “If I promote you will you be able to love me? or, ‘Will you be able to produce for me a child and if I promote you will you be able to do ABC ‘ (many others) and so in that due course, the female nurse will feel shy and accept in order to achieve her goals, thus a* ***sexual harassment in administrative*** ***way.*** *Another thing is about going for further studies which is not a case for all of them (the nurses) and in case the doctor has seen that the lady is beautiful or has satisfied him sexually, produced children for him, all this can force the manager to sign for her and he will freely give her what she want such as further studies.”*   - **Implied or explicit threats (Expression of an intention to inflict pain, injury, damage, or other hostile action on someone in retribution for something done or not done)**   *“It may be a consequence because we have different ways of winning, they may do it just to scare/* ***threaten*** *you or just to put you in a scaring mood such that you give in. It may be a strategy for someone. So, they can start with that and then others.”*   - **Implied/explicit threats, blackmail in performance appraisals**   *“A person can call you into the consultation room and you go there not knowing what he is going to tell you. You go there thinking he has called you for a special purpose and when you reach there, he starts telling you things of sex. And if this is your boss, he might tell you that* ***if you refuse, I will put other things on your report*** *that you have done this and that. When such a person reports you to the higher office, they will not mind if what he is saying is right or wrong and yet you have not done anything*.”   - **Performance appraisal is used by male supervisors as an opportunity to settle scores for refusal**   *“For me I opposed him and he even made late phone calls but if he could tell me something I could just divert him because I knew what I was supposed to do. So for him he reacted to it , he wrote a paper (appraisal) and put it in my file saying (she) does not work, does not do this and that (her chores)… the HR called me that this would* ***affect my career*** *as I had* ***not been absorbed in public service****.”*  *“When you want your boss to appraise your performance* ***you may have to give*** *in for appraisal to the in-charge or the head of department.”*  *“There are those who write a very* ***bad comment*** *on your appraisal. Appraisals are the worst; they wait for you at the time of the appraisal and harass you.”*  ***“Blackmail****- A supervisor implicating a subordinate in reports because she denied his sexual advances.”*   - **Retaliation/punishment/Making work/life difficult if sexual favors are not granted**   *“I have observed and I have even heard about it from the ladies that some senior member of staff-a male was trying to make passes to some female staff and when they did not respond, he became* ***hostile*** *to such an extent that they could not go to his ward*.”  *“The forms of sexual harassment I am familiar is especially between* ***superiors*** *more common among men harassing women. If some superior wanted a relationship and the subordinate does not want, the superior can start to treat that person in* ***unfriendly*** *way.”*  *“One time there was a scenario where a supervisor was involved in* ***transferring*** *a certain health worker to another facility simply because she had refused his sexual advances.”*  *“To explain more, the male superior will call a lady in to his office and tell her that he wants to have sex with her just like that. And when she refuses, then she will be* ***sacked*** *you know.”*  *“Then there is also another one where a female* ***ruined*** *the boy (i.e., a young male health worker). He reported it to my office as an in charge of the institution…The boy* *said- No, don’t take me to that unit because that woman there did A, B, C and it was physical sexual harassment…The boy* *reported but it was difficult to handle that case because she was an in-charge and elderly and she denied the case and we had to leave the case there. …so the boy was made to move away from that unit.*   - **Favoritism for those who submit to demands for sex**   *“Also conditions of your salary being worked upon after you have given sex, otherwise they* ***can deny you salary*** *for a whole year yet the person responsible for it demands for sex.”*  *“But sometimes it is economical because you have refused to give in to the sexual favors, you are* ***denied*** *some economic benefits- it could be allowances, field trip, salary reduction, transfer so that your economic chances are reduced depending on how you give in to the sexual favors or not. …There are people who rotate in workshops or trips. If it is field, she is the one, workshops- she is the one in charge of the money/accounts, she/he is on every list. … so you are denied of some economic benefits as a way of harassing you to give into a sexual demand.”*   - **Overt or subtle threats of dismissal or demotion if sexual favors are not granted** (NB: Only example of female on male coercion)   *“She used her office and tried to* ***threaten*** *me that if I don’t I will be chased from the work place, I am a man and in African traditional we are told to be strong but when it comes to* ***threatening*** *me because I didn’t* ***submit*** *in what you wanted because of a job and I have do your demands that is not okay for me.”* | **Physical**   - **Deliberate and unsolicited physical contact**/**unnecessary close physical proximity/invasion of space**   *The environment like when you are working in one room….* *You sit like sometimes our offices are few, so you sit congested so near to each other which hence attracts them.”*  *“If there is a workshop, they want to stay close to you. If it is residential, they will demand for sex while there.”*  *“Male colleagues* ***enter changing rooms*** *without knocking because they want to see female nurses’ nakedness.”*  *“There is also this scenario of* ***hugging.*** *Instead of hugging me, he just pulls you closer to him and holds you tightly. I may interpret this in a different way.’*   - **Non-consensual touching**   *‘“I have gotten a golden chance to have you” and he then tells you that “this is the best moment to express myself”. He will hold you tightly and squeeze you and when you try to* ***resist*** *he will use* ***force.****’*  *“He maybe illustrating something like hypertension and he used your body especially the bums and breasts to take advantage of* ***touching*** *them with* ***liberty.****”*  *“The forms of sexual harassment I am familiar is especially between superiors more common among men harassing women. If some superior wanted a relationship and the subordinate does not want, the superior can start to treat that person in* ***unfriendly*** *way,* ***touching*** *the person.’ between colleagues, for example the male touching the breasts of the female when she doesn’t want it-they are just working together-but this person keeps* ***touching without permission****.*  *“****Bad touches****. Someone can touch your* ***bums,*** *breasts, and other some other parts that are* ***unwanted and unpleasant****. Someone can come and touch on your* ***nose and chin****. “*  *Touching the person between colleagues, for example the male* ***touching the breasts*** *of the female when* ***she doesn’t want*** *it-they are just working together-but this person keeps touching* ***without permission.”***  *“When greeting some men, they can* ***tickle/scratch*** *inside the female’s* ***hand****”*.   - **Squeezing**   *“Squeezing. The way he squeezes you is not normal. He can even give a kiss on any part of your body.”*   - **Kissing**   *“Kissing- maybe kissing, but there is that intimate kissing that may be sexual. It is a sexual kiss, not merely kiss, a sexual peck, not merely peck.”*   - **Stalking** (to approach or follow in an insistent, apparently unobtrusive way)   *“There is also some of us where our staff can work at the night duty, someone can attempt to come and* ***attack*** *you while departing on your way from the facility back home which happened some time back but the person* ***fought*** *and escaped the tragedy. Somebody prepares to have you after continuously failing to have sex with you, so he will target you on your way back home at night.”*   - **Rape/sexually assault**   *“****Forced*** *sex, male colleagues force the females* ***against their consent****… The boss can call you in his office and force you to have sex with him”*  **Verbal:**   - **Belittling, objectifying language about a person’s appearance or body, verbal retaliation for refusing**   *“… And also making comments about the lady-he makes a pass at the lady but she responds in a negative way so he starts making negative comments about her body-to* ***belittle*** *her, to annoy her.”*  **Verbal (abuse)**   - **Taunting**   “*You are* ***ugly’****, ‘whom do you think is interested in you’ when he was actually interested in her. No it is sour grapes* “  *“Because they black mail,* ***threaten you, blame*** *you, and use* ***abusive*** *language and many others….*  *“So, words spoken- telling you how you are sexual, how you are not using your endowment, how you are not exploiting yourself for higher offices, abusing you, you are* ***ugly t****hat’s why you have no body loving you. The approach can be different- can* ***be abusive*** *or* ***praising*** *but you are* ***uncomfortable*** *with the comment. …….. It is psychological… In your mind you wonder- Am I stupid? Am I less of a woman? So it is* ***physical, sexual, and psychological****.”*  *“You are told how* ***beautiful*** *you are. Like that name calling- sweetheart, honey, virgin Mary- an older woman wonders why someone calls her a virgin Mary, because we have not seen you sleep around! You are told how you are “magulu gaamu” (meaning have glue between your legs,* ***can’t give in*** *for sex) - we wonder who sleeps with you, who parts those legs! But after all maggots will eat it-* ***you refuse*** *giving us.***”**  *“Sometimes, he can even use verbal words. Funny words like ‘Musawo’ (health worker), when are you giving me?”*   - **Repeated sexually-oriented comments about the body or appearance of a person**   *“And* ***intimidating words****, somebody comes and tells that you have very big buttocks and you think your husband is faithful to you?”*  *“Use of* ***vulgar language*** *like, ‘You are proud with your big buttocks.’ Give us your buttocks after all, it will not talk.”*  *“Anyway some talk like that to get sexual contact. When you* ***refuse*** *they start throwing those* ***bad*** *words,* ***abusive*** *words to you. Some can say, “you are just running with those buttocks of yours which are very useless.”*   - **Sexually explicit jokes, bantering, sexual innuendo or sexual words**   *“Using vulgar words which are not good for our mouth to pronounce. They tell you*  *stories about sex and ask you if you have visited your aunt (having pulled your labia)”/“They ask you “Did you to visit your auntie?”*  *“Sometimes when the nurse is helping a clinician or a medical officer- sometimes*  *when they are giving out anesthesia –they can just say “please can you pass me-*  *of course for the needle to penetrate, you need some force- so he may ask, ‘Has it*  *entered?’ Of course those are some of the jokes and you know ladies can respond*  *in a funny way*.”  *“Use of seductive …or sexual words”*   - **Persistent invitations to social activities after the person has made it clear they are not welcome**   *“Sexual harassment can also be* ***psychologica****l through continuous demand for sex or sexual favors. Can I take you out for dinner; will you escort me for the workshop? Do you think you will get married to an angel? Can’t you cheat on your husband? It is* ***unwanted*** *but* ***continuously*** *demanded for.”*   - **Sexually- colored remarks or compliments**   ***“Inappropriate*** *words like someone would say, you look* ***hot:*** *you are my size, not as a compliment but to entice somebody. “*  *“They can tell you face-to-face how they imagine you in their beds; whatever happens in bed, when people are having sex.”*   - **Sexist remarks**   *“Provoking you. Calling you* ***unqualified*** *when you refuse sexual advances.”*  **Written/Visual:**   - **Offensive letters, text or e-mail messages**   *“Pornographic films. Sharing and watching of pornographic films through mobile phones. After someone watching the pornographic film, he can send it to the other person.”*  *“Sending you romantic messages. Like “I love your lips”, “I love your eyes“…I love your thighs” …Your figure is sexy.”*   - **Photos or drawings of sexual images**   *There is also this one of Whatsapping, where you send someone a message. I have seen someone Whatsapping and send someone weird photographs and enticing messages, texts. Though the individual is* ***not willing****, but because of your enticing messages might feel enticed.”*  *“There are some people on Whatsapp-they send funny pictures and movies about sex. Such people who send such messages will want to know about your comments on the pictures or movie clips that they send you to see if you are interested.”*  **Gestural:**   - **Nods, winks, gestures with the hands, fingers, legs or arms, signs and other offensive**   *“Even gestures; someone can look at your breasts and then makes a sign on the breasts that ‘I want to suckle your breasts.”*  *“The high or flying kisses and winking of the eyes.”*  *“Some can even close one eye and open another at you (winking)”*  *Another form is for midwives in labor wards. The doctor is doing VE and he notices the structure and starts looking at you…You can see the doctor blinking at you and making gestures like trying to say that, after here….”*   - **Persistent leering at the person or at part of his/her body**   *“You may be working and when you raise your eyes, you find him staring at you”* |
