# Supplementary material for: Uganda’s response to sexual harassment in the public health sector: from “Dying Silently” to gender-transformational HRH policy
Source: Hum Resour Health. 2021 May 1;19:59. doi: 10.1186/s12960-021-00569-0 (PMC8087889; doi:10.1186/s12960-021-00569-0)
Supplement: Supplementary file 5 — Additional file 5: Two Cases of Secondary Injury after Reporting Sexual Harassment. [file 12960_2021_569_MOESM5_ESM.docx]

**Additional File 5:** **Two Cases of Secondary Injury After Reporting Sexual Harassment**

This section provides illustrations of victim-blaming and “secondary injury” to victims as a result of reporting or trying to report *quid pro quo* sexual harassment. The cases illustrate injury beyond the initial harm of sexual harassment, and why victims would be reluctant to report. These cases embody findings from Baugh’s ^^[[1]](#footnote-1)^^ research suggesting that a woman who attempts to make a claim about sexual harassment is “faced with the task of demonstrating to a male-biased, if not male dominated power structure the legitimacy of her complaint.... The woman claimant must make her case and hope for resolution in a system in which *her views do not predominate*.”

**Case 1:** The first case illustrates how coerced submission to the sexual demands of a hierarchical superior is called “consent” in spite of the power differential. A victim also does not report a boss’ *quid pro quo* sexual harassment for fear of being stigmatized by the rest of the staff. The victim who did not comply reported that she was ultimately imprisoned, and suffered trauma, health and family impacts. There are few alternatives in the face of the abuse of superior power.

| **Case 1: Interview with Key Informant National Level**  I: *So, in your experience, what type of person is the most common victim of sexual harassment?*  R: Women who are attractive- good looking women. Then those in the lower cadre- clerks, servants are usually the targets, young magistrates. What has fallen in my ears is that their immediate bosses (I don’t want to mention names) demand for sex when they go out for workshops and when they are in the field there. They do not demand but will ask- and because this is an immediate supervisor, these girls are constrained to give in because they fear the repercussions- promotions, posting to stable places. That is the challenge they face much as they may not want but go ahead to sleep with them. And these are magistrates- professionals. It is very common where men are in power- they use these powers to exchange sex for favors. *A woman was also sentenced because she refused to give in.*  I: *So, what are the consequences, impacts and harms of sexual harassment*?  R: It can actually flow over to the family of this girl. Because I know of one incident- one judicial officer who raped an office attendant and she became pregnant and family got disintegrated. It happened to my sister, she complained to her husband and he was not happy about it – of course about it causes friction within the family.  I: *Okay, if we look to the impacts like to the individual, work atmosphere and organization. So if we start like with the individual and you have told me a person’s family can disintegrate, affects morale, what is another negative impact to the individual*?  R: The person is *traumatized* for example where this woman was *sentenced* because she refused to give in. She was traumatized by the whole experience- she was in prison.  I: *Okay, we can go to under reporting. Studies on sexual harassment in other countries- say this is very common. Do you think this is the case in Uganda?*  R: It is. Very few report and those who report are very bold. In judiciary we had those two cases- two people came forward and report. There is another magistrate who used to do that but was *interdicted* on other reasons and was removed from the service. And the moment that case reaches one of our superiors, they will investigate and take action and will not keep quiet. Unfortunately very few come to report.  I: *Some say they don’t know how to report, is that applicable to our workplaces?*  R: Yeah, you know with these this issue of reporting, people don’t want to talk about sensitive issues. Somebody can’t stand out and say, someone one asked me for sex and I gave in. Or sometimes you have already given in and so the person will say you already gave in, so you accepted. Why are you complaining? So, it was *consensual and not harassment*. There are very few who know that even just being harassed is sexual harassment. And if it goes to the level of sex then you have already consented.  I: *Then what about feelings of shame?*  R: The shame comes in when this girl has given in.  I: *What about fear of being ostracized or being exposed or being blamed for it?*  R: I don’t know about that fear of being blamed. Like I pointed they may *fear to be stigmatized* or being *pointed a*t that, that girl reported so and so. So in other words she would be punished for it by the rest of the staff for reporting the boss. I know during the 80’s –during the peak of HIV, so many girls were harassed and many of them got *infected*. I remember there were some of these office attendants- they wanted the job and this guy was in charge of recruitment… |
| --- |

**Case 2:** The second case illustrates many of the gender dynamics operating in the reporting of sexual harassment. In this case, the victim faced *quid pro quo* sexual harassment, but did not comply. Sexual harassment was normalized. When the target reported, she was faced with unfair legal evidentiary requirements, where an administrative solution was sought. Doubts about her experience and her report, minimizing or rationalizing the experience, counter-accusation of nonperformance, and intimidation occurred in retaliation for reporting. The risky group- and gender dynamics of a committee was also illustrated, and in fact, the harasser did not take the process seriously enough to show up. The reporting victim finally lost her educational program, job and marriage.

| **Case 2: Key Informant National Level**  I: *Now, on reporting, Studies of sexual harassment in other countries indicate that there is under-reporting of sexual harassment and it is very common.*  R: They have called it under-reporting but here we have *non-reporting*. Before you go to underreporting, here we have non- reporting. In fact, that’s the most common in Uganda. I don’t know how many times you have been harassed and you have not reported to your husband, bosses, to the police. You are harassed in taxi, Taxi Park, on the bus, in office, hospital. And it’s like you brush it away and move on like that. *Non- reporting is common because that’s the way we have been brought up.* *We excuse the men for harassing men; we see it as part of life.*  I: *Why do you think we look at it as part of life?*  R: *Socialization*- the way we have been brought up by society. The gender lenses of society- we have not grown up seeing these things as evil but as part of life of what a man or woman are expected to do. It is a gendered culture but not gender responsive. From day one we are told expect that. A Muganda will even say “*ekijja omanyi, kinyaga bitono, ekiddukano tekirya byenda” (meaning whatever calamity comes when you are aware, the consequences are not grave).* So it comes when you expect it but you take it as a job risk acceptable- that when you are walking you will knock your toe against a stone- you but continue, you don’t cancel the journey. Or you are walking through a forest and you step on a thorn- you remove it and continue with the journey. So, that’s the way we have grown up. It has been accepted in the dimensions of life. Even if you are married and it happens, somebody tell you that marriage is not a bed of roses! When you are being introduced, married, oriented at a job- you will be told not to go reporting simple things etc. You will be appraised against your social behavior that you should be outgoing.  I: *Other than the social set up, what could be cause? For you have said it is non-reporting.*  R: If you are my supervisor and you are refusing to mark my work for example and you are pegging it to a sexual favor, I should have another supervisor without necessarily proving *beyond reasonable doubt*. There is a famous case in [ ] University which was out under the carpet but which is think is a live case. I don’t know if Dr. [ ] can accept being interviewed. She did her undergraduate degree at [ ]. During that time the lecturer got interested but she *managed to dodge*. She accepted the harassment but she never gave in to sexual intercourse. She passed very well and was retained as a teaching assistant. She was given a master’s program and unfortunately she was put under the supervision of the same doctor who was harassing her. To cut the long story short- the supervising Dr. [ ] …. a senior doctor removed the drugs she was using for her study from the official fridge at the hospital and took them to the fridge at his sitting room. And this student was forced to cross the road, pick the drugs to continue with her drugs on the children. Every time she would go to his sitting room, she would be subjected to sexual harassment of a peck, kiss or hands touching the boobs, hugs, touching the bums, asking her why she can’t give in, if she will be married to an angel- and she was married to a fellow lecturer. But she continued dodging. One day she says she was given a drink- the doctor said “let’s have a wine but she refused, then a juice – she tastes 1, 2, 3 sips and she feels dizzy. She read something on the wall and said excuse me doctor I left something at the clinic- she ran back to the clinic and slept for 4 hours and she knew the juice had been laced and she could have been raped. So she resolved to speak out and the human resource did not help her. She said “Aaah haven’t you ever heard of this song – *dolly w’omwana akaaba*? (meaning “a child’s doll”). She gathered courage to go to the vice-chancellor.... He said *are you sure,* *has it happened*? What did he do- *only touched you*? Then the doctor explained. Sshhhhh *only that*! He *only touched yo*u? Don’t you know doctor so and so (names a female doctor) and doctor so and so. They told us that’s *how their love began*. Why are you so obsessed with this? *Is that a matter worth reporting to me*? If you are serious putting it in writing. So he was “eeh! You are serious? We are constituting a *disciplinary committee* to *try this* matter.” But the men’s club said this girl is bringing allegations against a senior medical personnel and she should be put to task to *prove beyond reasonable doubt*. So *she becomes the accused*. They i*nterdict* her and gave her about 7/9 counts- *insubordination, bringing false allegations and she is tried*. The doctor was never put to task yet these are allegations which should have been put to Doctor [ ]. She was told “You have failed to prove your case beyond reasonable doubt so we are expelling you”. She said I don’t mind if you are expelling me, but what I am telling you is the truth. By the way he was marking your work *but you failed to submit your work in time and that’s why you are doing to find cheap ways of getting marks without working for them*. She said, I am not looking for a criminal conviction- *I am only seeking for an administrative action*. But all I want is a change of my supervisor. They said first make an apology but she was stubborn and refused to make it. Doctor knows, we have been in *these committee meetings 9 times*- where is the doctor? He has never been to these committees and I am the only one testifying against him, and it is you people taking on the harassment. The doctor *never appeared in any of the committees*, she was thrown out and her master’s program was cancelled, told to return the laptop and the books and was expelled from lecturing. She came to me (she is a relative). I took her to the Inspector General of Government- by then Justice [___]. She told her story and [person reported to] cried in her office. Then she said let us kneel down and pray – and we knelt down and prayed against those big men that harass young doctors etc. She constituted a commission to carry out investigations. Then the investigator opened up and said “*You know something fishy has come up- my boss is not accepting my findings* etc. You brought me a girl who is telling lies- a *non-performer*, recruited through back door, failed to do the work. Then a phone call comes in- “Yes, they are here, I can’t let down a leader of government business”. So my mind told me she was talking to a business leader and that is the prime minister– orders from above! He said now you have to apologize and pointed at the President’s portrait and said that’s the brain child behind [ ] University and the donors are saying if there is sexual harassment, they are cutting off aid until there is an anti-sexual harassment policy in place and until these lecturers are expelled. We have to save face of the university, of the president. The doctor cried and said “Madam if you can’t help me” then don’t abuse me”. [ ]’s wires go off and said “[ ], - tell your cousin to apologize”. But I said she is a doctor, she is an adult who brought the story. I can’t order to apologize. She lost a job, masters and then later lost the marriage because the husband had to choose between losing the job. The marriage went on the rocks and she was thrown out. I *disagreed proving beyond reasonable doubt* because when it comes to administrative matters. The IGG’s report should have been like a postmortem. So burden or standard of proof is very, very important for reporting and non-reporting for stopping the impunity or not stopping it. Or underreporting – what are the viable remedies? People are looking at only the criminal remedy of imprisoning somebody, but it could be a fine, apology, reconciliation, transfer within a department or out of a ministry, a re-allocation of duties, putting somebody in an office which is glassed, regulation of work times, reporting mechanism on daily basis where there is confidentiality and secrecy because sexual harassment are not issues for every Tom, Harry and Dick to discuss…. |
| --- |

1. Baugh, S.G. On the Persistence of Sexual Harassment in the Workplace. Journal of Business Ethics (1997) 16: 899. Pp.31-33. [↑](#footnote-ref-1)
