# Supplementary material for: Uganda’s response to sexual harassment in the public health sector: from “Dying Silently” to gender-transformational HRH policy
Source: Hum Resour Health. 2021 May 1;19:59. doi: 10.1186/s12960-021-00569-0 (PMC8087889; doi:10.1186/s12960-021-00569-0)
Supplement: Supplementary file 6 — Additional file 6: Regional, Ethnic and Gender Stereotypes. [file 12960_2021_569_MOESM6_ESM.docx]

**Appendix 6: Regional, Ethnic and Gender Stereotypes**

Asked in mixed-group FGDs and IDIs whether victims of sexual harassment had any particular characteristics. However, some respondents mentioned that women from east and central Uganda are more sexually harassed than those from northern Uganda. Reasons given were that women from central Uganda are perceived as *Dot.com* *girls*, more modern/urban than those from the northern region. Another perception was that women from some parts of Uganda are more sexually appealing or “easy”. Note the conflation of sexuality with sexual harassment, until one respondent makes a distinction:

“*Yeah - the man knows that this one, the Senga (aunties) should have influenced her to have those labia pulled and most men say they like it. Even the way these girls from central and near east dress, they are always in short uniform, painted herself, she has decorated herself definitely the male partners get friendly to them very fast compared to these northern ladies.” (IDI Female)*

*“There is also a saying that a single woman out there will fall for a northern man because they are appreciated. The northern man likes the light- skinned woman so a Muganda lady will suffer because they are always lighter than the northerners.” (FGD, Mixed Group)*

**Box 1: Ethnicity, gender and sexual harassment (Mixed Sex FGD)**

| **Does a person’s ethnic group affect whether they will be targeted with sexual harassment?**  R5: To some extent yes. We have grown up with some beliefs that ladies from western Uganda are perceived to be good in bed besides having a lot of water.  R4: They are water-logged.  (Laughter)  **M: Which people are those?**  R4: Banyakole women…..  R5: The men from Northern and eastern Uganda are also perceived to be good in bed…  **M: What do you have to say about ethnic groups?**  R5: Then our sisters from Busoga are perceived to give in easily. So if I target those ones, I will find it easy to sleep with them.  R6: I hear even the Batooro women can be targets because of that perception so they can be very much targeted.  **M: And let us concentrate on the health sector?**  R5: It does not matter whether she went to school or not, what is important is that she is coming from some of those regions.  **M: So people from such regions are more targeted because of their ethnicity?**  R4: Very much.  R2: Baganda women are also perceived to be well brought up and so they have that respect, they are disciplined and this makes them want to go with the Baganda.  **M: Will that then be sexual harassment?**  R2: If the victim does not want it, then it is sexual harassment. |
| --- |
