# Supplementary material for: Uganda’s response to sexual harassment in the public health sector: from “Dying Silently” to gender-transformational HRH policy
Source: Hum Resour Health. 2021 May 1;19:59. doi: 10.1186/s12960-021-00569-0 (PMC8087889; doi:10.1186/s12960-021-00569-0)
Supplement: Supplementary file 7 — Additional file 7: Patient Harassment by Health Workers. [file 12960_2021_569_MOESM7_ESM.docx]

**Additional File 7: Patient Harassment by Health Workers**

Evidence of the sexual harassment of patients emerged unexpectedly from the Phase 1 FGDs. For example:

“*In addition to what she has said, I have heard a story of a health worker who was supposed to examine a patient but instead of going to the part the patient was complaining about, he went directly to the private parts. He straight away went for vaginal examination instead of the headache the patient was complaining about.” (FGD, Female)*

“*Yes. When a person is doing a medical examination, they prefer to do what we call ‘bad touch’. A patient may be complaining about a different condition but again you go and start doing a different examination of touching.” (FGD, Male)*

*“To add to that, there is a story I heard that a patient went for an examination and the clinician told the patient that he needed to put the thermometer in her private part to get the right temperature. The patient came out and told the people outside what the doctor did.”* (FGD, Mixed)

*“There was a health worker who used to ask for sexual favors from female patients whenever they would come into his clinic. Every woman who would come into his clinic would be forced to have sex with this officer.”* *(FGD, Male)*

“*This one concerns the health workers. In most cases, when the patient comes, instead of the health workers asking those questions which are related to the problem/sickness, he starts to ask irrelevant questions…’Ooh where are you coming from, ooh! You are a good lady, do you have a husband?’ Things of that nature.”* *(FGD, Male)*

These reports were followed up in interviews with 16 In-Charges in facilities where this behavior was reported in FGDS. Irrelevant or unnecessary vaginal and breast exams and “bad touching” were reported to be the most common forms of sexual harassment of patients by health workers, though forms also included displaying a patient’s nude body or body parts during clinical exams, and as well as sexual assault, including rape [See **Table 1]**. Some patients were believed to comply with unwanted touching and coercion, while others were reported to react violently. However, the informants noted that victims *rarely* reacted by reporting the health worker to police, hospital administrators or others for fear of negative consequences. Systematic research is needed to estimate the prevalence of patient sexual harassment in health systems and to understand its dynamics, effects and consequences.

| **Table 1: Patient Harassment by Health Workers (From Follow-Up Interviews with Facility In-Charges)** | |
| --- | --- |
| **Types of Harassment** | **No of Interviews Out of 16** |
| Asking irrelevant sex tinged questions to patients | 7 (43.7%) |
| Irrelevant vaginal /breast examinations, follow up appointments | **15 (93.7%)** |
| Prolonged greeting and touching of breasts | 2 (12.5%) |
| Communication with the eyes | 1 (6.25%) |
| Direct requests for sex (e.g., during cervical cancer screening) | 7 (43.7%) |
| Rape/having intercourse with patients | 6 (37.5%) |
| “Bad touching” | **12 (75%)** |
| Use of vulgar/ obscene language | 4 (25%) |
| Suggestive gestures like winking, kiss showing interest in patients | 3 (18.7%) |
| Use of verbal words to patients like you look nice, nice bums | 3 (18.5%) |
| Use of phones for calling and messages to patients | 2 (12.5%) |
| Stalking of patients by health workers/ Asking for contacts for asking for sexual relation | 4 (25%) |

Some facility level informants viewed (indecent) dressing and other aspects of female sexuality mainly as an excuse to justify male sexual harassment, and placed responsibility on the harasser. A national-level key informant noted that harassing patients results in perceptions of poor service quality, tainted reputations of MOH facilities and providers and ultimately, in the nonuse of government health services. “Indecent dressing” was cited as having no role in the following scenario described by one informant:

*“There was one case scenario - this was not an employee but then it was someone seeking for a service in one of the public places and it happened to be* ***a nun****. She went to this person and this person without shame turned to this person and started demanding for sexual favors from the nun. Of course, nun made an alarm that attracted attention and probably the church should have started from there but…no decision was taken.” (National-Level Key Informant)*

**Sexual exchange/transaction:** Follow- up interview respondents reported that sexual transactions are expected of patients by some clinicians. In some instances, patients are believed to *use the existing system* with an expectation that sex is exchanged for better and faster treatment and medicines. This finding was consistent with, and parallels, findings of *quid pro quo* sexual harassment by the supervisor of his health worker supervisee, where the transaction revolves around sex for a job/promotion/training. In both cases, the transaction appears to fit the definition of “sextortion.” [See **Additional File 2.1,** Concepts and Definitions]

**Secondary injury:** Some patients are believed by In-Charges to give in to the harassment, and some, to react violently. However, they noted that victims *rarely* react by reporting the sexually harassing health workers to police, hospital administrators or others. One facility in-charge recounted a story in which a young woman who had been sexually assaulted by a clinician complained to her family and village chief, who asked her not to report further, lest the village lose the one health service provider they had. Clients are perceived to be unaware of their rights, and to fear refusing a clinician’s sexual demands. The responses of in-Charges also suggested that patients would experience *secondary injury* resulting from reporting, including: an expectation of victim-blaming; heightened scrutiny; fear of a counter-accusation; lack of support from others; fear of inaction; not knowing the outcome of reporting; and most of all, fear of retaliation by the harassing health worker who might ignore the patient or her children in the future and deny them health services and medicines.
