# Supplementary material for: Uganda’s response to sexual harassment in the public health sector: from “Dying Silently” to gender-transformational HRH policy
Source: Hum Resour Health. 2021 May 1;19:59. doi: 10.1186/s12960-021-00569-0 (PMC8087889; doi:10.1186/s12960-021-00569-0)
Supplement: Supplementary file 8 — Additional file 8: How the MOH Took the Study Results into Account. [file 12960_2021_569_MOESM8_ESM.docx]

**Additional File 8:** **How the MOH Took the Study Results into Account**

The research results were disseminated within the UMOH and among stakeholders in 2017 and used in 2018 to develop *Guidelines to Implement the Policy on Prevention and Response to Sexual Harassment*, which were posted on the UMOH website: Uganda Ministry of Health. *Guidelines to implement the policy on prevention and response to sexual harassment*. 2018. <http://library.health.go.ug/publications/gender-based-violence/guidelines-implement-policy-prevention-and-response-sexual>. The UMOH passed them into force in early 2018 and led a national multi-sectoral launch with extensive media coverage later that year.

The MOH *Guidelines* designers, who were drawn from key government ministries, took study results into account in several ways. First, the UMOH *Guidelines* used research evidence to define what sexual harassment was. For instance, the examples of sexual harassment in Ugandan health workplaces were derived from the study participants’ (who had been targets) descriptions of their experiences. This shifted the power to define sexual harassment to the target of harassment. This was *transformative* in that it challenged current norms, valorized the target’s experience and not the perpetrator’s intention, shifted power relations and allowed targets of harassment to “reclaim the narrative” [71]. The principle of an integrated community approach to prevention takes the burden of ending sexual harassment off the shoulders of the individual target and places it on the organizational community and its leadership. The *Guidelines* also used the research evidence to design a reporting system with multiple entry points and both informal pathways and formal administrative mechanisms, to supplement the criminal law whose agents (police, courts) may not have previously been responsive or accountable to victims.

The *Guidelines* also used findings on the contributing factors at individual, organizational and societal levels, as well as the abuse of power by men at higher levels in the organizational hierarchy, to communicate an ecology of sexual harassment perpetration. This shifted the focus away from individual causation which was important given the tendency to suggest (as another Ugandan policy did) that sexual harassment could be stopped by enforcing stricter dress codes, which inappropriately “blames” the victim’s clothing for their harassment. Instead the *Guidelines* emphasized zero tolerance, gender equality, community responsibility and respect for human rights. The foregoing was integrated into an UMOH employee training module with posters and a brochure with accessible messages distributed during training.

The *Guidelines* describe a vision, principles and approaches that explicit include equal opportunity, nondiscrimination and respect for human rights, linking gender equality and the elimination of sexual harassment. **Table 1** is an excerpt from the *Guidelines*.

| **Table 1: Excerpts from the Uganda MOH** **Guidelines to Implement the Policy on Prevention and Response to Sexual Harassment** |
| --- |
| **“Vision:** A health sector that ensures *zero tolerance* to sexual harassment, upholds gender equality and human rights, and improves productivity of the workforce and its clients.” |
| **“Introduction and Background:** Sexual harassment prevention and response is crucial to good health systems governance and human resources management leading to achieving equal opportunity, nondiscrimination and gender equality in the health workforce…It is essential for addressing health worker attrition, job dissatisfaction and low productivity, victimization, poor performance, accidents, unwanted pregnancies, interpersonal conflict and absenteeism among others.” |
| **“Guiding Principles:** The implementation of the guidelines will be guided by the following principles:  **Human Rights**  The right to a safe and healthy working environment is a universally and fundamentally recognized human right to which men and women are entitled. Therefore, elimination of discrimination and sexual harassment is very key in realization of this right.  **Equal opportunity and non-discrimination**  A health sector where women and men have equal opportunity for employment, pay and promotion; equal access to and control over resources and benefits at the workplace without discrimination on the basis of gender, age, ethnicity or disability.  **Right of Association**  The right of association under the Labour Act No. 7 of 2006, particularly the right not to interfere with, restrain or coerce an employee in the exercise of his or her rights guaranteed under the Act and other related laws  **Confidentiality**  The guidelines recognize that sexual harassment leads to stigmatization and victimization of victims and whistle- blowers. All interventions to implement these guidelines shall ensure confidentiality, privacy and respect of the victims and whistle blowers. All service providers and duty bearers shall ensure that information about the victims shall only be divulged with their consent in order to ensure their safety and security.  **Gender Equality**  A health sector where women and men have equal chances to realize their full rights and potential to participate in the workforce, contribute to health development, and benefit from its results. This includes sensitivity to the effects of unequal power relations.  **Survivor-Centered Approach**  A survivor-centered approach necessitates that response and intervention to the assault centers on the voice and consent of the survivor. The substantive participation of the survivor should be considered at every stage of the prevention and response process.  **Integrated Community Approach**  Significant change is possible when all community members take collective responsibility to eliminate the root causes of gender-based violence and specifically, sexual harassment: gender inequality, abuse of power, and lack of respect for human rights. This approach creates individual responsibility on men, women, boys and girls, policymakers, community leaders, religious leaders, and other stakeholders, to prevent and respond to sexual harassment…In addition, men as potential perpetrators of sexual harassment must be involved in combatting sexual harassment through community sensitization and promotion of healthy attitudes as champions of sexual harassment prevention.  **Multi-sectoral Approach**  The guideline shall promote integrated approach and collaboration in the sector departments, institutions, units and facilities as well as other relevant Ministries, Departments, Agencies (MDAs) and Local Governments.  **Integrity in investigations**  All members involved in investigation will have high credibility, sensitivity and technical competency to handle such grievances”. |
| **“Prevention Strategies…**“Furthermore, this approach is community-oriented and demands collective responsibility from the entire community, including men and women, to prevent sexual harassment. Public health interventions are grouped into three prevention categories based on when the intervention occurs… |

The UMOH *Guidelines* provided policy directives to health sector employees related to two particular abuses of power and trust documented by the study: First, the *Guidelines* state that “*whereas there is no Ugandan law that forbids civil servants and other categories of workers working in the same institutions from being involved in a consensual romantic relationship and/or marriage, good practices from other organizations suggest that the two work in separate departments; and require that they should not be in a supervisee/supervisor relationship to avoid conflict of interest or its appearance.”* p.5]. Second, the UMOH *Guidelines* responded to evidence of sexual abuse and harassment of patients through explicit behavioral guidance: *“In line with the client’s charter, any medical examination shall be done in the presence of a third party. In case of a child patient below 12 years old, the examination should happen in the presence of their guardian. In case of an adolescent (13 years to 17 years) or an adult patient (18 years and above), the examination should happen in the presence of another health worker.* *Private parts’ examination to be done with full written consent of the patient/client or the parent/guardian and co-signed by the health worker in the presence of a third party.”* [p. 25.].

“The UMOH *Sexual Harassment Prevention and Response Guidelines* took a human-rights and multi-sectoral approach to zero tolerance. They specify governments’ administrative and legal obligations as duty bearers to operationalize the 2012 Sexual Harassment Regulations in work policy and environments, in which sexual harassment and retaliation are declared unlawful, including the UMOH itself [See Section 6.1 of Guidelines].  These include establishing informal and formal grievance processes as well as referral to law enforcement; articulate the sanctions available to Rewards and Sanctions Committees to discipline and deter perpetrators of sexual harassment [e.g., suspension of employment or certification to practice, demotion or termination of employment from the public sector]; and the responsibilities and authority of Service Commissions to take administrative action. The *Guidelines* constitute administrative policy to operationalize the employment law, but make it clear that internal MOH processes do not preclude legal redress. To this end, for example, Section 6.11 instructs the Uganda Police Force to operationalize the 2012 Sexual Harassment Regulations by investigating complaints of sexual harassment, collecting evidence, arresting and detaining the alleged perpetrators in accordance with the law, referring the victim for psycho-social, medical and other support services, and giving evidence in court.” According to CEDAW Recommendation 35, the government of Uganda has the option to undertake *ex officio prosecution to bring alleged perpetrators to trial in a fair, impartial, timely and expeditious manner and imposing adequate penalties.*

The UMOH put in place formal and informal grievance processes and trained supervisors and frontline staff in the study districts. The SHRH project ended before the use and effectiveness of the new reporting channels were evaluated. However, in 2021, a workplace climate improvement survey will be conducted in 30 new health districts of Uganda’s Eastern region and results used to improve health facility leadership and governance capacity to make and implement by-laws and policies on sexual harassment prevention and response.
